# Genomic profiling of a collection of patient-derived xenografts and cell lines identified ixabepilone as an active drug against chemo-resistant osteosarcoma

**DOI:** 10.1186/s13046-025-03440-5

**Published:** 2025-07-08

**Authors:** Maria Cristina Manara, Francesca Bruzzese, Laura Formentini, Lorena Landuzzi, Laura Pazzaglia, Maria Antonella Laginestra, Marianna Carrabotta, Maria Serena Roca, Federica Iannelli, Laura Grumetti, Laura Addi, Alessandro Parra, Camilla Cristalli, Michela Pasello, Alberto Bavelloni, Francesca Carreras, Francesca Ruzzi, Giuseppe Bianchi, Marco Gambarotti, Alberto Righi, Alfredo Budillon, Pier-Luigi Lollini, Katia Scotlandi

**Affiliations:** 1 Laboratory of Experimental Oncology, IRCCS Istituto Ortopedico Rizzoli, via di Barbiano 1/10, Bologna, 40136 Italy; 2 Experimental Animal Unit, Istituto Nazionale Tumori IRCCS Fondazione G. Pascale, Naples, Italy; 3 Department of Medical and Surgical Sciences (DIMEC), University of Bologna, Bologna, Italy; 4 Experimental Pharmacology Unit, Istituto Nazionale Tumori IRCCS Fondazione G. Pascale, Naples, Italy; 5 Clinica Ortopedica e Traumatologica III a Prevalente Indirizzo Oncologico, IRCCS Istituto Ortopedico Rizzoli, Bologna, Italy; 6 Department of Anatomy and Pathological Histology, IRCCS Istituto Ortopedico Rizzoli, Bologna, Italy; 7 Scientific Directorate, Istituto Nazionale Tumori IRCCS Fondazione G. Pascale, Naples, Italy; 8 IRCCS Azienda Ospedaliero Universitaria di Bologna, Bologna, 40138 Italy

**Keywords:** Osteosarcoma, Patient-Derived-Xenografts, Drug resistance, High-throughput drug screening, Ixabepilone

## Abstract

**Background:**

Osteosarcoma (OS) shows a multitude of genetic and chromosomal abnormalities together with large biological heterogeneity. These features limited the identification of novel drugs to treat patients with metastases and/or chemo-resistant tumors. The purpose of this study was to create additional resources for drug screening by generating patient-derived xenograft (PDXs) and PDX-derived cell lines that reflect the spectrum of OS heterogeneity.

**Methods:**

PDXs were derived from OS collected at diagnosis, surgical resections, or metastases. PDX-derived cell lines were also established. Targeted DNA sequencing and digital PCR were applied to identify major genetic alterations. High-throughput drug screening by using a library of 2880-FDA approved compounds and conventional MTT assays were performed to identify the most effective drugs against in vitro and in vivo growth of chemo-resistant OS.

**Results:**

Targeted DNA sequencing demonstrated alterations in the most commonly amplified oncogenes, such as *MYC*,* CCNE1*,* DDR2*,* CDK4*,* MDM2*, and *AURKA.* Recurrent deletions and SNVs were found in *TP53*,* CDKN2A*,* RB1*,* PTEN*, and* VHL.* Copy number variant (CNV) alterations in PDXs, PDX-derived cell lines and xenografts developed from cell lines (CDX) correlated very well with those observed in the matched original human tumors. Drug screenings identified and repurposed five compounds with efficacy against chemoresistant OS. In this context, we prioritized ixabepilone as a drug capable of inducing tumor regression in mice.

**Conclusions:**

We enriched the scientific community with additional, molecularly characterized OS models to be used for testing novel therapies and supported the inclusion of ixabepilone into treatment plans for chemoresistant OS.

**Supplementary Information:**

The online version contains supplementary material available at 10.1186/s13046-025-03440-5.

## Background

Treating osteosarcoma (OS), a highly aggressive primary tumor of bone, is still challenging. Despite the tumor is relatively rare (0.2% of all malignant tumors), the real obstacle lies in its extreme intra- and inter-tumor variability. This variability has greatly hindered the identification of key oncogenic drivers involved in the disease’s onset and progression, thereby significantly limiting the development of new therapeutic drugs. Over the last three decades, the therapy and the survival rate of patients with OS remained substantially unchanged with only one drug approved by the European Medicines Agency for the treatment of patients with a diagnosis of localized OS. Mifamurtide, a fully synthetic lipophilic derivative of the muramyl dipeptide that is encapsulated into liposomes, was authorized based on the phase 3 INT-0133 trial [1], which showed a significant benefit of the drug for overall survival. However, the mechanisms of action and the real benefit of mifamurtide for patients with nonmetastatic OS remain controversial and its use is still limited [2]. Thus, improving the therapy of OS patients still represents a major unmet clinical need.

OS displays peculiar epidemiologic, genetic, and biological features. Epidemiologically, OS shows a bimodal age incidence distribution, with more than half of cases arising in children or adolescents/young adults (AYA) and a second smaller peak in the elderly [3]. OS in elderly patients is often considered a secondary neoplasm attributed to sarcomatous transformation of Paget disease of bone or of some other benign bone lesions. In contrast, approximately one-fourth of children or AYA with OS had a highly penetrant germline mutation in a cancer-susceptibility gene [4], indicating distinctions in the pathogenesis and underlying biology among OS. Histologically, OS can be divided into several subtypes (i.e. osteoblastic, chondroblastic, etc.), likely reflecting the fact that OS is thought to derive from stem cells to osteoblast precursors with different levels of differentiation [5]. Genetically, several studies have shown the complexity and the instability of the genetic make-up of OS, which displays a multitude of cytogenetic abnormalities (*for a review see* [6, 7]. However, DNA sequencing also reveals the possibility to classify OS into different subtypes with distinct clinical prognosis, indicating the potential of future precision medicine [8–12]. To achieve this goal, the availability of appropriate experimental models that accurately recapitulate the epidemiologic, genetic, and clinical heterogeneity of OS is highly desirable. In recent years, patient-derived tumor xenografts (PDXs), which are reported to preserve the main features of the original tumors [13–15] and PDX-derived cell lines have been established for a wide variety of tumors, including OS [8, 16–18] and variably used to test novel therapies. We contribute to enlarging these collections by generating 30 PDXs in NOD scid gamma (NSG) mice and six PDX-derived cell lines, fully clinically annotated and molecularly defined by targeted genome sequencing and droplet digital PCR (ddPCR). A PDX-derived cell line established from the biopsy of a patient who did not respond to standard of care treatment and had a rapid dismal outcome, and carrying *MYC*,* DDR2* and *CCNE1* gene amplification in addition to *RB* gene loss was considered for high-throughput drug screening using a library of 2880 FDA-approved compounds. The screening led to the selection of 44 potentially active drugs assignable to 7 different mechanisms of action. The efficacy of 7 drugs, representative of each class of agents, was validated against the other five PDX-derived OS cell lines with similar or different molecular features, guiding to the identification of ixabepilone, a drug that alter the dynamics of the microtubules [19], as the most effective one.

## Methods

### Acquisition of patient samples

Fresh tumor samples were obtained, under sterile conditions, from surgical specimens and grossly examined by pathologists (MG or AR) for the presence of viable tumor tissue. Whenever the sample size and quality were assessed to be adequate, the available material was divided into fragments and processed as previously described [17]. Briefly: (a) tissue to be implanted into immunodeficient mice to generate PDX was placed in Iscove’s modified Dulbecco’s medium (IMDM, Euroclone #ECB2072L) supplemented with 10% fetal bovine serum (FBS, Euroclone, #ECS5000L) and antibiotics (penicillin, streptomycin, Merck, #P4333), hereafter referred to as complete medium; (b) tissue for genetic and molecular studies was flash-frozen in liquid nitrogen and stored at -80 °C; (c) tissue for histopathology and immunohistochemistry was fixed in a 10% buffered formalin solution. All specimens were reviewed by pathologists (MG and AR) and the diagnosis of OS was confirmed. All the clinical annotations of the patients were retrieved from the register of the Musculoskeletal Tumor Biobank of the IRCCS Rizzoli Orthopedic Institute.

### Mice and PDX setup

Immunodeficient NOD Scid gamma (NSG) (NOD.Cg-Prkdcscid Il2rgtm1Wjl/SzJ, RRID: IMSRJAX:005557), mice were originally obtained from Charles River, Italy and bred under sterile conditions in animal facility (P-LL). To generate PDXs, fresh tumor specimens of approximately 4 mm^3^ were implanted subcutaneously (s.c.), at the level of the trans-scapular brown fat of 5-11-week-old male NSG mice, within an average of 1–2 h after surgery. If not implanted on the day of surgery, tumor fragments were stored in liquid nitrogen in freezing medium (90% FBS, 10% Dimethyl Sulfoxide, DMSO, Merck, #D2540) and thawed on the day of implantation. Tumor growth was monitored with calipers at least twice weekly until it reached a maximum volume of 2.5 cm^3^, at which time the mouse was euthanized by CO_2_ inhalation and cervical dislocation. The tumor was collected, and a careful necropsy was performed to assess overt metastatic spread. Tumor fragments were implanted into NSG mice for PDX propagation, or distributed in cryovials, in 90% FBS + 10% DMSO, for viable storage in liquid nitrogen. For molecular studies, tumor samples were snap-frozen in liquid nitrogen and stored at -80 °C, while for histopathologic evaluation they were fixed in a 10% buffered formalin solution.

### PDX and patient-derived cell lines generation

Tumor samples obtained from PDXs were minced into small pieces and placed in complete medium in 60-mm dishes (Becton Dickinson,#353002) and incubated at 37 °C in a 5% CO_2_ humidified atmosphere. When the outgrowth cultures formed a confluent monolayer, the cells were subcultured after enzymatic treatment with 0.05% trypsin-EDTA (Merck, T4174) and maintained in vitro for at least 10 passages before being processed for in vitro and in vivo studies. Cell lines were authenticated by short tandem repeat (STR) analysis (CLA service of Eurofins Genomics) in comparison with the profile of the original surgical specimen and, the PDX. The human origin of in vitro cultures was confirmed by PCR analysis using the mouse feeder cell quantification kit (Diatech Labline, #RR290) according to the manufacturer’s instructions [17].

Six PDX-derived cell lines (designated as PDX-OS#2-C, PDX-OS#16-C, PDX-OS#19-C, PDX-OS#25-C, PDX-OS#29-C, and PDX-OS#30-C) were genetically characterized and used for in vitro and in vivo studies. The patient-derived human OS cell lines Saos-2 and U-2OS were obtained from the American Type Culture Collection (1992). The OS patient-derived cell lines IOR/OS9, IOR/OS10, IOR/MOS, and IOR/SARG were established at the Laboratory of Experimental Oncology, Rizzoli Orthopedic Institute, and previously characterized [20, 21]. The MTX-, and CDDP-resistant variants of U-2OS cell line were established by exposing the sensitive U-2OS cell line to stepwise increasing concentration of each drug, as previously described [22, 23].

To avoid cross-contamination between cell lines and outgrowth of faster-growing clones in long-term cultures, all cell lines were stored in liquid nitrogen until use. When in culture, they were maintained in complete medium and incubated at 37 °C in a humidified atmosphere containing 5% CO_2_ for approximately 8–12 in vitro passages (equivalent to 2–3 months) before being discarded. Whenever necessary, replicates were started from the same batch of frozen vials. Cells were regularly tested for mycoplasma contamination (MycoAlert Mycoplasma Detection Kit, #LT07-418, Lonza) and periodically authenticated by STR PCR analysis (last control July 2024).

### DNA extraction

OS human samples along with their corresponding PDX have been evaluated by pathologists (AR and MG) to verify that more than 70% of cells are of neoplastic origin. DNA was extracted from patient’s tumor, PDX, PDX-derived cell lines xenografts developed from cell lines (CDX) and a pool of 6 human peripheral blood mononuclear cells (hPBMC) from healthy donors purchased from Lonza (hPBMCs Lonza CC-2702 19TL064725), using DNAzol (Thermo Fisher Scientific, # 4452222) and its quality was evaluated by Implen-GO NanoPhotometer N50-GO (IMPLEN) before being further processed.

### DNA targeted sequencing, alignment, and quality control

Targeted amplicon sequencing was performed using the Archer^®^ VariantPlex^®^ solid tumor panel (Archer DX). The targeted panel allows Single Nucleotide Variants (SNV) in 63 genes and Copy Number Variants (CNVs) for 44 genes (Suppl. Figure 1). First, we assessed the quality of DNA input with Archer PreSeq DNA Quality Control (QC) assay by calculating the number of amplifiable genomes available in each sample, according to the Archer PreSeq DNA Calculator Assay protocol (Archer DX). According to the Archer PreSeq DNA Calculator Assay protocol (Archer DX), DNA was fragmented, amplified using specific primers and quantified by KAPA Universal Library Quantification Kit (Kapa Biosystems, Roche). The paired-end libraries (2 × 150 bp) were then sequenced on the NextSeq500 platform (Illumina) following the manufacturer’s instructions. After quality control, FASTQ files were aligned to the human GRCh37/19 reference by BowTie2 [24] and Burrows-Wheeler Alignment (BWA) algorithms [25].

### Copy number analysis of targeted amplicon sequencing

Copy number alteration was inferred from targeted amplicon sequencing data in all samples against a pool of 6 hPBMC from healthy donors. CNV kit software (v0.3.5) (RRID: SCR_021917) with default parameters [26, 27] was used. Exploiting a BED file that lists the genomic coordinates of the baited regions and aligned sequencing reads (BAM files) we firstly calculated the sequencing coverage of the targeted regions for each tumour and normal sample. Then, we combined all normal samples into a pooled reference. The read depths of the tumour samples were normalized, corrected for GC content and repetitive sequences, and individually compared to the reference, obtaining a final table of log_2_copy ratios. The HAAR segmentation algorithm (wavelet-based method) was run on the log_2_ratio values to infer discrete copy number segments. Copy number segments were annotated to genes and segments were considered as deletions if log2 ratio < − 1.7, and as amplifications if log2 ratio > 1.7. Spearman’s correlation analysis based on the normalized depth coverage of CN genes in panel was used to assess similarity among OS patients, PDXs, PDX-derived cell lines and cell-derived xenografts (CDX). Correlograms were generated using CorrPlot R package [28].

#### Single nucleotide variant calling

SNVs, insertions and deletions (indels), were analyzed with Archer^®^ Analysis v6.0.4 software (Archer DX). Molecular barcode (MBC) adapters were used to count unique molecules. In particular, variant detection was performed with LoFreq [29] and freebayes algorithm implemented in Archer Analysis using default settings [30]. Ensembl Variant Effect Predictor (VEP) [31] was used for functional annotation of variants, including stop-gain single nucleotide variants, frameshift and indels. All the mutations found were filtered based on the following criteria: Alternative Observations ≥ 5, Unique Alternative Observations ≥ 3, gnomAD AF ≤ 0.05, AF Outlier P Value ≤ 0.01. Individual variants were manually evaluated, retaining only those variants that were reported in the COSMIC database v98 (latest release May 2023).

### Droplet digital PCR (ddPCR)

To validate copy number alterations affecting the *MYC*, *CDK4*,* DDR2*, *CCNE1*, genes we used Droplet Digital PCR (Bio-Rad laboratories) and Taqman Copy number Assays (ID: hs01764918:cn, hs01071103_cn, hs05780148_cn; hs00452646_cn respectively) TaqMan Copy number RNAseP human as reference assay (Applied Biosystems, 4403326. Briefly, 4ng of DNA from OS patients, PDXs, and PDX derived cell lines and CDX were processed according to manufacturer’s protocol. PCR reaction mixtures were loaded into a DG8 cartridge (Bio-Rad laboratories) with 70µL of droplet generation oil through the Bio-Rad automated droplet generator system (Bio-Rad laboratories), which produced approximately 20000 oil droplets/reactions. The generated droplets were then read using a QX200 droplet reader (Bio-Rad) for the determination of total fluorescent. The data were analyzed with QuantaSoft version 1.7.4.0917 (Bio-Rad laboratories). Based on the absolute quantification of the target molecules per reaction, the gene copy number was determined as the ratio between target genes and endogenous; the ratio was multiplied by 2 to express the absolute copy number value. Amplification was defined as CNV > 3 gene copies [32].

### Tumorigenic and metastatic ability of PDX-derived cell lines and in vivo drug testing

To assess tumorigenic ability of OS PDX-derived cell lines, 2 × 10^6^ cells were injected s.c. in the hind limb of 3 NSG mice per group. Tumor size was measured using calipers twice a week and tumor volumes were calculated according to the formula π[√(a×b)]^3^/6, where a = maximal tumor diameter and b = tumor diameter perpendicular to a. Mice developing s.c. tumors were sacrificed when the tumors reached a volume of 2.5 cm^3^. To assess experimental metastasis, 2 × 10^6^ cells were injected intravenously (i.v.) in a lateral tail vein of 5 NSG mice per group. Mice developing experimental metastases were sacrificed when initial signs of metastatic growth appeared that was at 6-9-13 weeks from i.v. cell injection for fast, intermediate, or slow growing tumors, respectively. The mice were subjected to an accurate necropsy. The lungs were perfused with black India ink; lung and liver metastases were counted using a dissection microscope, and any other metastatic lesion in other sites (mainly adrenal glands, interscapular adipose tissue and lymph nodes) was recorded, collected, and fixed in a 10% buffered formalin solution.

For the study of in vivo drug activity, 12-39-week-old male NSG mice received s.c. injection of 2 × 10^6^ PDX-OS#30-C cells. After 14 days, when tumors started to reach a volume around 50–100 mm^3^, mice were randomized (8–9 mice per group) to receive vehicles or drugs. Ixabepilone was administered at the dose of 6 mg/Kg by i.v. injection for three administrations at 4-day intervals (q4d x3). Ixabepilone powder (MedChemExpress) was dissolved in DMSO to 5 mg/ml for stock solution and diluted in saline, at 0.45 mg/ml, immediately before i.v. injection in a lateral tail vein. Homoharringtonine was administered at the dose of 2 mg/Kg by intraperitoneal (i.p.) injection [33], once a day, four days a week, for 2 weeks, for a total of eight administrations. Homoharringtonine (MedChemExpress) was dissolved in DMSO to 5.5 mg/ml for stock solution and diluted in Phosphate Buffered Saline -Tween 80 (Merck, P4780) 5%, at 0.30 mg/ml, immediately before i.p. injection. Tumor size was measured as described above. The number of mice enrolled in each test group was calculated using a power analysis tool (available from: https://www.sealedenvelope.com/power/binary-superiority) to have an 80% chance of showing, with a 5% significance, a 50% of success in the experimental group. Blinding to assess the outcome of in vivo experiments was not done.

Body weight was checked weekly and before each drug injection. Mice were sacrificed 30 days after cell injection. Accurate necropsies were performed, tumors were collected for histopathological and molecular analyses as described above.

### Immunohistochemistry

Tissues were fixed in 10% buffered formalin, processed routinely, and embedded in paraffin. Serial 3-µm-thick paraffin sections, mounted on precoated slides, were processed according to standardized automated procedures (Ventana Medical Systems inc., RRID: SCR_013652) and immunostained with the following antibodies: anti-MDR1 P-glycoprotein (PGP) (ABCB1), clone JSB-1 (Monosan, #MON9011-1, RRID: AB_221060, 1:50 dilution), anti-CD68 antibody, clone KP-1, mouse monoclonal, prediluted; (Ventana, Roche, RRID: AB_1158191), anti-Ki67, clone 30 − 9, rabbit monoclonal (Ventana, Roche Cat# 05278384001, RRID: AB_2631262,prediluited). Antigen retrieval pretreatment was performed with Tris-EDTA, pH 8.00, at 95 °C for 20 min. Staining was performed using the UltraView Universal DAB Detection Kit (Ventana Medical Systems, 760 − 500, RRID: AB_2753116). For evaluation of CD68 staining, samples were considered infiltrated with human macrophages, when more than few scattered macrophages were present in between the tumoral cells.

The PGP-positive tumor specimens were graded from 1 to 3 according to the distribution of positivity and the degree of immunostaining of the cytoplasm as follows: A score of 1, scattered positive cells (involving less than 10% of the sample) and weak immunostaining; a score of 2, diffuse positivity (more than 10% of the sample) and weak immunostaining; and a score of 3, diffuse positivity (more than 10% of the sample) and strong immunostaining. Specimens with no staining or a score of 1 were considered negative, while specimens with a score *≥* 2 were considered positive [34].

Appropriate positive and negative controls were included in each run, and all stained sections included non-tumor mouse cells, such as endothelial cells, myopericytes, and fibroblasts, which were always negative. For morphological analysis, slides were stained with hematoxylin, rehydrated, and coverslipped.

Histological and histomorphometry analyses were carried out with a digital pathology slide scanner with a resolution of 0.5 μm/pixel (Leica Aperio AT2 scanner, Aperio Technologies, Vista, RRID: SCR_021256), using ImageScope software (v12.4.3, RRID: SCR_014311) for slide viewing and analysis.

### High-throughput drug screening– one-shot screening assay

High throughput screening was performed into twenty-five 384-well flat-bottom plates (Cell Carrier Plates, PerkinElmer). The drug library comprised 2880 compounds from HY-L022_FDA-Approved Drug Library by MedChem Express. Compound liquid handling steps were performed using a Micro Lab-STAR Liquid Handling Workstation (Hamilton). Cells were trypsinized with 0.05% trypsin-EDTA, counted using the TC20 Automated Cell Counter (Biorad, RRID: SCR_025462), resuspended in media to the desired concentration, and then seeded into 384-well plates for screening using Micro Lab-STAR Liquid Handling (Hamilton). Cell seeding density was optimized to achieve approximately 70% confluence at the end of the treatment period in vehicle-treated conditions. Seeded cells were incubated for 24 h prior to drug treatment for 4 days at a single dose of 0.1µM and 1µM in monoplicate of each 2880 compounds along with two positive controls (doxorubicin 1µM, Merck, D1515 and panobinostat 5µM, Bio Vision), DMSO (1% or 0.1%) and vehicle. Then, the plates moved back to the incubator and after 96 h the cell viability was assessed.

### High-throughput dose response assay

The concentration of drug able to kill the 50% of cells (IC_50_) was evaluated for the 7 compounds selected from one-shot screening assay. Micro Lab-STAR Liquid Handling by Hamilton was employed to seed cells (2,000 cells/well) into two 384-well flat-bottom plate (Cell Carrier Plate, PerkinElmer). Subsequently, the plates were transferred to the incubator for further processing. After 24 h after seeding, all plates were treated simultaneously by Micro Lab-STAR Liquid Handling, with a titration of compounds in quadruplicate along with two positive controls, doxorubicin (1µM) and panobinostat (5µM) and vehicle (water and DMSO). Specifically, the titration curve for azlocillin (sodium salt), tirbanibulin (dihydrochloride), olverembatinib, hydroxycitric acid (tripotassium hydrate), homoharringtonine, ixabepilone, clofarabine, was 100nM, 50nM, 25nM, 12.5 nM, 6.25nM, 3.156nM, 1.156nM. Then, the plates moved back to the incubator and after 96 h the cell viability was assessed.

### High-throughput drug screening data analysis

To ensure the reliability of the results, internal quality control was conducted. This involved calculating the Z factor for each plate. Z-factor (Z-f), is a measure of statistical effect size and calculated as the means (µ) and standard deviation (s) of both positive (p) and negatives (n) controls:

Z-f = 1–3(sp + sn)/|mp-mn| (Suppl. Figure 2).

Acceptable Z-f value ranges between 0.5 and 1. The plate is invalidated if Z-f value is below 0.3. The Z-f value can never exceed 1 [35]. All plates met this criterion, as evidenced by Z-f greater than 0.5, indicating successful quality control. To evaluate the percentage of cell growth given by each compound, the mean luminescence recorded in the plate measured at time 0 was subtracted to the experimental plates value, following the equation: %cell growth = Compound X value/Ctrl value*100).

To calculate IC_50_ and R2, the concentrations of each compound were initially transformed to log (X) and normalized, and nonlinear regression was performed using the GraphPad Prism 7.0 software (GraphPad Inc, RRID: SCR_002798).

The targets of the 44 selected drugs were identified in Drug Bank. The networks between identified targets were obtained using Ingenuity Pathway Analysis (IPA) software (GeneGo Inc., RRID: SCR_008653), which visualizes proteins as hubs and the relationships between proteins as edges.

### Cell viability assays

For high-throughput drug screening, cell viability was determined using the Cell Titer-Glo^®^ Cell Viability Assay (Promega, G7570). The homogenous CellTiter-Glo^®^ Luminescent Cell Viability Assay quantifies the amount of ATP present to ascertain the total number of viable cells in culture. An amount of 40 µl of this single reagent was added directly to cells grown in serum-supplemented media as part of the homogeneous assay technique, according to the manufacturer’s instruction. The uniform “add-mix-measure” method caused cell lysis to produce a luminous signal whose intensity was directly related to the amount of ATP in the sample. Ten minutes after reagent addition and mixing, luminescence readings were obtained using BioTek Cytation 5 Cell Imaging Multi-Mode Reader (Agilent-BioTek, RRID: SCR_019732). The luminescence (lum) was recorded by Gen5 Software (RRID: SCR_017317).

Validation analyses were performed in the PDX- and patient-derived cell lines and chemoresistant cell variants obtained from the commercial U-2OS cell line in our laboratory [22, 36]using the TACS^®^ MTT Cell Proliferation Assay kit (Trevigen, 4890/25/K). Cells were plated into 96-well plates (10,000 cells/well) in standard medium. Twenty-four hours after cell seeding, cells were treated for 96 h with the following drugs: homoharringtonine (# HY-14944), ixabepilone (# HY-10222), azlocillin (sodium salt; #HY-B0529A), tirbanibulin (dihydrochloride; #HY-10340 A); hydroxycitric acid (tripotassium hydrate; #HY-W009156) and olverembatinib (#HY-15666), all purchased from MedChem Express. Working dilutions of all drugs were prepared immediately before use. Where applicable, cells were also treated with dimethyl sulfoxide (DMSO, Merck #D2650) as control.

Cell growth was determined using resazurin-based PrestoBlue reagent (Invitrogen, A13262), according to the manufacturer’s instructions. Briefly, the PrestoBlue solution (10 µL) was added to each well, and the plates were then incubated for an additional 2 h. The plates were then directly read on an Infinite M200 photometer (Tecan Group Ltd) at a wavelength of 600 nm. Data were graphed using GraphPad Prism 7 software (RRID: SCR_002798) Three replicates per tested concentration and at least three independent experiments were performed.

To evaluate drug efficacy in 3D conditions, we assessed spheroid formation in anchorage-independent conditions after seeding 10,000 to 100,000 cells per dish in 0.33% agarose (SeaPlaque, Lonza) with ixabepilone (3nM-30nM) or homoharringtonine (10nM-100nM), alongside a 0.5% agarose underlay. Colonies that contained more than 10 cells were counted with an inverted microscope 14 days after seeding (Nikon Diaphot).

### Statistical analysis

GraphPad Prism (version 6.0 software; RRID: SCR_002798) was used for statistical analysis. Statistical analyses were performed using parametric tests for data with normal and symmetric distributions (one-way ANOVA); otherwise, nonparametric tests were used (Kruskal-Wallis and Dunn post hoc tests for unpaired data or two-way ANOVA multiple comparison with Geisser-Greenhouse correction and Mann-Whitney U test for unpaired two-group data). Values are expressed as mean ± SD. Kaplan–Meier progressive-tumor-free survival curves were compared using the Mantel–Cox log-rank test.

## Results

### OS PDXs and PDX-derived cell lines closely mimic the biological and genetic heterogeneity of the patient’s tumor of origin

**Fig. 1:**
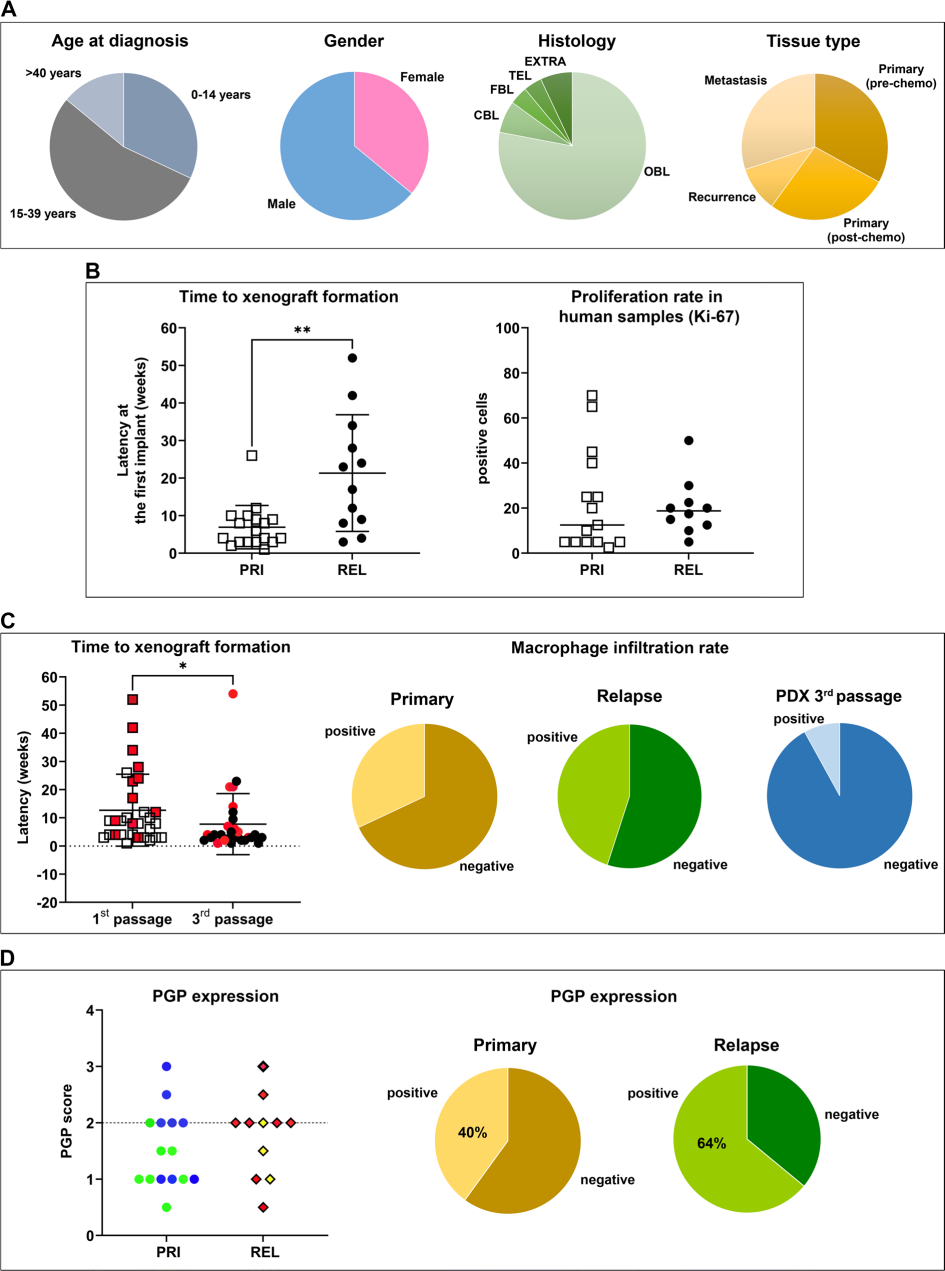
**A**, Clinical-pathological characteristics of OS patients enrolled in the study (28 patients/30 PDX-OS models) (OBL, osteoblastic; CBL, chondroblastic; FBL, fibroblastic; TEL, telangiectatic; EXTRA, extraskeletal). **B**, (*left*) PDX latency at first implantation in relation to tumor sample type, in primary tumors (PRI) versus relapses (REL) (including local recurrence and metastasis) (**p < 0.01 by Mann-Whitney U test). (*right)* Percentage of Ki-67-positive cells in PRI versus REL (not significant by Mann-Whitney U test). **C**, (*left*) PDX latency at the first and third in vivo passage (*p < 0.05 by Mann-Whitney U test). Red symbols indicate REL. (*right)* The proportion of samples showing macrophage infiltration among samples deriving from patients’ primary tumors (32% positive) or relapses (45% positive) and PDXs at passage 3 (8% positive) are shown as pie charts. Macrophage infiltration rate in human samples and corresponding PDX at third passage is shown as a pie chart. **D** Expression of P-glycoprotein (PGP) score calculated as in PRI versus REL. Symbols in blue and green refer to pre- and post-treatment in PRI, respectively; symbols in yellow and red refer to local recurrence and metastasis samples in REL, respectively. The proportion of PGP protein and negative samples in PRI (40% of positive samples) and REL (64% of positive samples) is shown as pie charts

Tumor samples were collected directly from patients at the time of biopsy or surgery and vital tumor fragments were implanted into the trans-scapular brown fat of 5–11-week-old NSG mice to generate PDXs. A total of 30 PDXs deriving from 28 OS patients were successfully engrafted and were available for this study. In two OS cases we received more than one specimen from the same patient, which included in one case, primary tumor before treatment (biopsy) and after neo-adjuvant chemotherapy (primary tumor resection post-chemotherapy), and in the other case, two distinct lung metastatic lesions. In keeping with our previous observations [17], successful engraftment was obtained in 39% of the implants. The clinico-pathological features of the patient’s tumors of origin that developed PDX are reported in Fig. 1A. In general, PDXs were obtained from pediatric patient tumors (*n* = 9, 32%), AYA patients (*n* = 15, 54%), and patients older than 40 ys (*n* = 4, 14%). The majority of PDXs were derived from male patients (*n* = 18, 64%). Osteoblastic OS (*n* = 22, 78%) was the most common histological subtype, with all the other subtypes being represented, including the very rare telangiectatic and extra skeletal OS variants. Eighteen PDXs (60%) derived from localized primary tumors (10 from untreated biopsies and 8 from tumors after preoperative chemotherapy), 12 PDXs (40%) derived from relapses (3 from local relapses and 9 from metastatic lesions in the lung).

After implantation of the surgical specimen in mice, the time required for the appearance of a sizeable tumor (latency time) was variable, ranging between 1 and 52 weeks. Differences were not related to patient age or gender or histological subtypes (Suppl. Figure 3). PDXs from primary localized tumors developed faster than those from local recurrences or metastases (Fig. 1B), although the proliferation rate of the original tumors, as indicated by the percentage of Ki-67 positive cells, appeared to be similar in primary and in relapses (Fig. 1B). When PDXs were maintained in mice for up to three in vivo passages, latency times converged to 4–5 weeks in all the models (Fig. 1C). This shorter latency was particularly evident in relapses and could be explained by the progressive loss of the human immune infiltrate [37, 38]. Indeed, the percentage of samples showing human tumor-infiltrating macrophages, the most abundant immune cells in OS [39], was higher in tumor tissues from relapses, which showed longer latency, compared to human samples from primary tumors and to a PDX after three in vivo passages where the human infiltrate almost completely disappeared (Fig. 1C), thus suggesting that human macrophages that are co-implanted along with tumor tissues might contribute to restrain tumor growth rate at the first implant in the mouse. To verify whether and at which extent our collection of PDXs is representative of chemo-resistant tumors, we performed immunostaining of PGP which is encoded by the multidrug resistant (MDR-1) gene and is known to have a critical role in OS [34, 40]. Evaluation was possible in 26 human tumor samples (15 primary tumors, 8 of which were naïve biopsies and 7 were tumor resections after preoperative treatment; 11 secondary lesions) and corresponding PDXs. The concordance between expression in tumors and matched PDX models was 86%. The protein was overexpressed in 40% of PDXs from primary tumors (6/15) and in 64% (7/11) of PDXs from secondary lesions (Fig. 1D; Suppl. Figure 3). Among the PGP-positive primary tumors, 5 of them were naïve biopsies, indicating that our collection represents either tumors with innate or acquired features of chemoresistance. All PDXs retained the morphological features of their respective patient’s tumor, including the production of neoplastic bone and the presence of anaplastic cells, over multiple passages (Suppl. Figure 4) and histologic concordance was confirmed by pathologists in all cases.

**Fig. 2:**
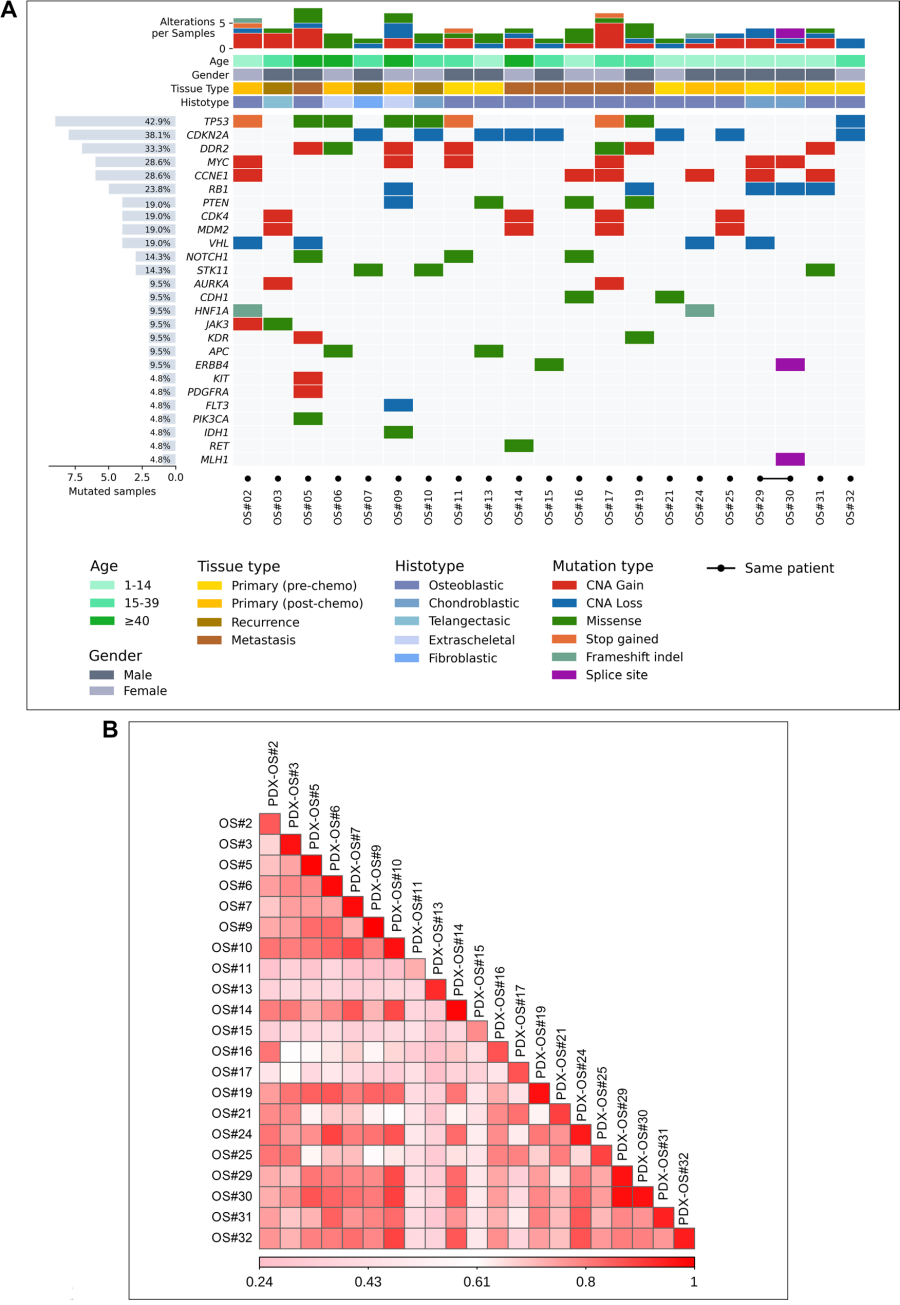
Identification of genomic alteration in human tumor samples and corresponding PDXs. **A**, Heatmap visualization of gene alterations in 21 samples from 20 OS patients. Copy number gain/loss was defined as log2 ratio segments log2 > 1.7 and log2<-1.7, respectively. The upper plot summarizes alterations per sample and clinicopathologic characteristics of the samples. The left bar graph shows the percentage of altered genes across human samples. At the bottom of the plot, samples from the same patient are labeled as linked circle indicators. Each column represents a single sample. **B**, Spearman’s correlation analysis of CNV in all human tumor samples compared to the pairing PDXs (log2 ratios for all evaluable genes on the Archer VariantPlex solid tumor panel) (range rho = 0.83–0.98). For all patients, the corresponding PDX correlated best with the original human sample

Targeted DNA sequencing was performed on 21 snap frozen tumor samples from 20 patients and the corresponding 21 PDXs (at 2nd or 3rd in vivo passage). In keeping with previous reports [8, 41], OS and PDXs demonstrated heterogeneous patterns of genetic alterations (Fig. 2 and Suppl. Figure 5). We found that the most amplified oncogenes in the human original tumors were *MYC* (6/21, 29%), *CCNE1* (6/21, 29%), *DDR2* (5/21, 23% carrying gain CNV plus 2 cases with missense mutations). In addition, we found amplifications in *CDK4* (4/21, 19%), *MDM2* (4/21, 19%) and in *AURKA* (2/21, 9%). Recurrent deletions and SNVs were found in *TP53* (9/21, 43%), *CDKN2A* (8/21, 38%), *RB1* (5/21, 24%), *PTEN* (4/21, 19%), and *VHL* (4/21, 19%) (Fig. 2A, Suppl. Table 1).

CNV alterations in PDXs (Suppl. Figure 5) correlated very well with those observed in the matched tumors (range rho = 0.86–0.96, Spearman test) (Fig. 2B), confirming the genomic similarity of the xenografts to the patient’s tumors. Amplification of *MYC*, *CCNE1*, *DDR2* and *CDK4* were also confirmed by ddPCR (Suppl. Figure 5).

From 6 PDXs we generated PDX-derived cell lines. All the cell lines derived from PDXs of pediatric or AYA patients, modeling different stages of the disease: one derived from a naive biopsy, three from post-treatment primary tumors and two from lung metastatic lesions (Table 1a). All the donating patients died for disease progression. Thus, these are experimental models of very aggressive, therapeutically challenging tumors. Particularly, the PDX-OS#29-C and PDX-OS#30-C cell lines derived from the same patient at different time of disease progression (PDX-OS#29-C from naïve biopsy and PDX-OS#30-C from surgical tumor specimen after preoperative chemotherapy).

**Table 1a:** Osteosarcoma PDX-derived cell lines, patient characteristics, genomic alterations and biological features. * ddPCR validation; ** only ddPCR results

Cell line ID	Patient				Cell line						
	Age	Sex	Met	status	Origin	Tx staus	Site origin	Genomic features	PGP % of positive cells (MFI)	Spheroids (soft agar)	DXR IC50 (ng/ml)
PDX-OS#2-C	11	female	yes (during treatment)	dead	primary	treated	femur	*MYC* gain*, *CNEE1* gain*, DDR2 gain**,*TP53* stop gained	24.2 (82)	336.5 ± 40.9	298.5 ± 88.8
PDX-OS#16-C	30	female	yes	dead	metastasis	treated	lung	*CDH1* missense, NOTCH1 missense	13.7 (7.2)	265 ± 29	69.8 ± 0.6
PDX-OS#19-C	9	male	yes	dead	metastasis	treated	lung	*DDR2* gain*, *PTEN* loss, *TP53* missense, *RB1* loss	18 (2)	143 ± 12	36.9 ± 3.8
PDX-OS#25-C	12	male	yes	dead	primary	treated	femur	*MDM2* gain, *CDK4* gain*, *CDKN2A* loss	0.7 (0.7)	80 ± 7	59.8 ± 31.4
PDX-OS#29-C	14	male	yes	dead	primary	naive	femur	*MYC* gain*, *DDR2* gain**, *CNEE1* gain*; *RB1* loss	37.7 (280)	573.8 ± 14.9	410.5 ± 54.3
PDX-OS#30-C	14	male	yes (during treatment)	dead	primary	treated	femur	*MYC* gain*: *DDR2* gain**; *CNEE1* gain*; *RB1* loss	49.5 (200)	675 ± 29.2	168.3 ± 59.9

**Table 1b:** Tumorigenic and metastatic ability of osteosarcoma PDX-derived cell lines after subcutaneous (s.c.) or intravenous (i.v.) injection of 2 × 10^6^ cells in NSG mice

Cell line ID	Tumorigenicity (s.c. injection)			Metastatic ability (i.v. injection)						
	Median time to sacrifice (weeks)	Incidence (%)	Median Latency (weeks, range)	Median time to sacrifice (weeks)	Lung metastases		Liver metastases		Other metastatic sites (kidney, adrenal, interscapular fat pad)	
					Incidence (%)	Median (range)	Incidence (%)	Median (range)	Incidence (%)	Median (range)
**PDX-** **OS#2-C**	11	3/3 (100%)	3 (3–8)	14	0/5 (0%)	0	0/5 (0%)	0	0/5 (0%)	0
**PDX-** **OS#16-C**	15	3/3 (100%)	7 (6–8)	13	2/5 (40%)	0 (0–17)	0/5 (0%)	0	0/5 (0%)	0
**PDX-** **OS#19-C**	16	3/3 (100%)	6 (6–7)	13	5/5 (100%)	2 (1–11)	0/5 (0%)	0	0/5 (0%)	0
**PDX-** **OS#25-C**	23	3/3 (100%)	17 (17–17)	13	0/5 (0%)	0	0/5 (0%)	0	0/5 (0%)	0
**PDX-** **OS#29-C**	7	3/3 (100%)	4 (4–4)	9	1/5 (20%)	0 (0–1)	5/5 (100%)	20 (16–25)	4/5 (80%)	4 (0–9)
**PDX-** **OS#30-C**	5	3/3 (100%)	2 (2–2)	6	5/5 (100%)	3 (2–5)	5/5 (100%)	45 (42–68)	5/5 (100%)	2 (1–8)

**Fig. 3:**
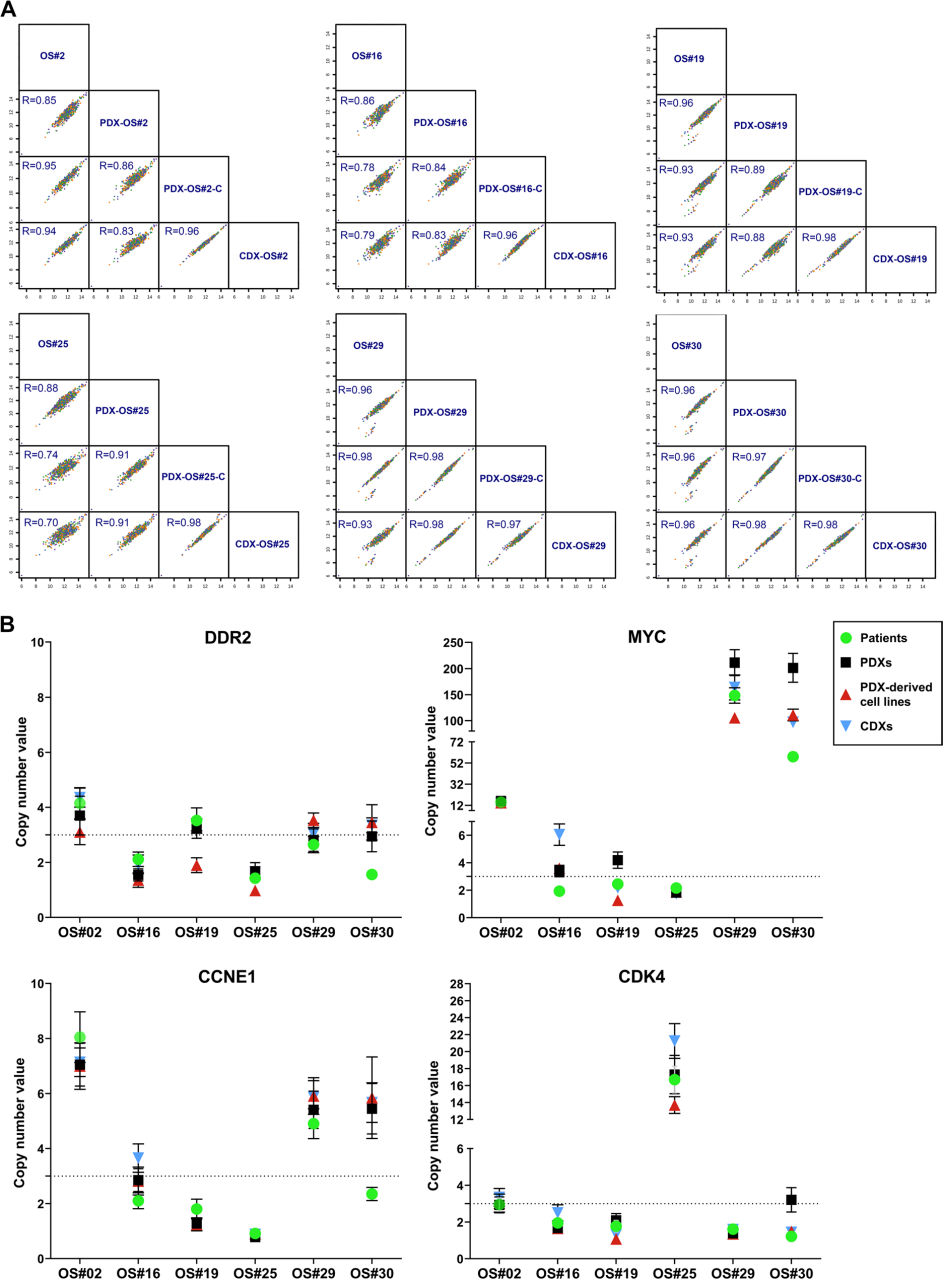
Copy Number Variant (CN)V analysis. **A**, Pairwise Spearman correlation analysis of CNV among patient’s tumor, PDX, PDX-derived cell line and CDX samples (log2 ratios for all evaluable genes on the Archer VariantPlex solid tumor panel) (range rho = 0.70–0.98). **B**, Validation of gene gain by ddPCR. Each marker represents a CNV measurement from a single ddPCR well of ∼ 20,000 droplets. Error bars indicate Poisson 95% confidence intervals for each copy number determination. Amplification was defined as the detection of CNV > 3 gene copies

These models represent an extremely aggressive form of OS which was completely unresponsive to standard treatments (histological response to chemotherapy below 60%) and led patient to death in 8 months from diagnosis. Targeted DNA sequencing confirmed the high correlation in terms of CNV among patient’s tumor, PDX and matched PDX-derived cell lines (range rho = 0.70–0.98, Spearman’s correlation test) (Fig. 3A). Amplification of *DDR2*, *MYC*, *CCNE1*, and *CDK4* gains was validated by ddPCR (Fig. 3B). As for tumors and PDXs, each cell line presents a peculiar set of major genetic alterations (Table 1a). PDX-OS#2, PDX-OS#29-C and PDX-OS#30-C cell lines are positive for PGP expression and had a lower sensitivity to doxorubicin, the leader drug in the OS treatment (Table 1a). All the six lines grew in anchorage-independent conditions but with different efficiency (Table 1a). Similarly, the ability of OSPDX-derived cell lines to form xenografts in NSG mice was highly variable (Table 1b). The latency of xenograft formation after subcutaneous (s.c.) injection of 2 × 10^6^ cells ranged from 2 to 17 weeks. All the xenografts derived from the cell lines (CDXs) were analyzed for genetic alterations by DNA targeted sequencing and Droplet Digital PCR (ddPCR) (Fig. 3A and B), showing the maintenance of the genetic features of the original tumor samples, PDXs and cell lines. The three cell lines with major amplification of *MYC* (#2, #29, #30) exhibited faster growth rates (Table 1b), among these the PDX-OS#30 gave rise to measurable tumors in two weeks. Besides strong *MYC* amplification, this cell line also presented modest gain of *CCNE1* and *DDR2* together with loss of *RB1*. In contrast, the PDX-OS#25-C cell line, which is less efficient in developing s.c. tumors displayed gain of *CDK4* and loss of *CDKN2A*. With respect to metastasis, none of the cell lines were able to develop spontaneous metastasis in our s.c. experimental conditions. Thus, the six PDX-derived cell lines were intravenously (i.v.) injected to study experimental metastasis. Mice were sacrificed after 3 months, with the sole exception of PDX-OS#29-C and PDX-OS#30-C, which confirmed to be the most aggressive cell lines, forming lung and liver metastases in 100% of mice and leading to sacrifice for ethical reasons after 9 and 6 weeks from cell injection, respectively. Overall, the in vivo behavior of these cell lines was quite variable and, as for human tumors, it remained difficult to link aggressiveness to a specific set of genetic or biological features. The other *MYC* amplified cell line PDX-OS#2-C, that formed local tumors quite rapidly, was unable to develop experimental metastasis; the PDX-OS#19-C cell line, which displayed alterations of the oncosuppressors *RB1*,* TP53* and *PTEN* but no oncogene amplification, developed tumors and lung mets in 100% of the animals, while the PDX-OS#25-C cell line carrying MDM2 and CDK4 gain and CDKN2A loss, but no *MYC* and *RB1* alterations, gave rise to slow growing s.c. tumors and was unable to form metastasis. (Table 1b).

### High-throughput and conventional drug screenings identified Ixabepilone as an FDA-approved drug with relevant efficacy in OS

**Fig. 4:**
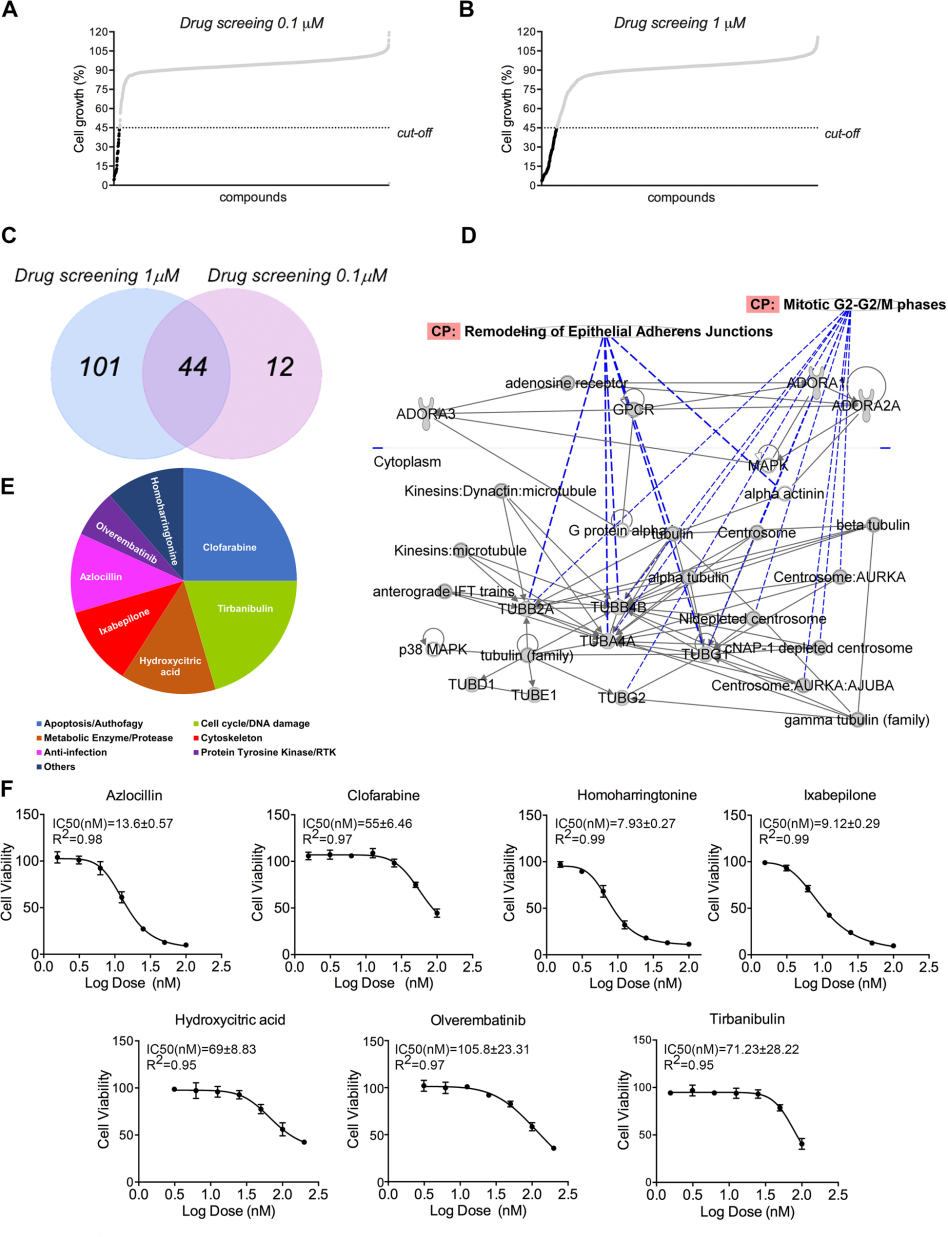
High-throughput drug screening results of 2880 FDA-approved compounds. **A**, Screening results at dose of 0.1μM. 56 compounds showed a cell growth ≤ 45%. (cut-off). **B**, Screening results at dose of 1μM. 145 compounds showed a cell growth ≤ 45% (cut-off). **C**, Venn diagram of overlap of effective compounds in screening performed at 0.1μM and 1μM. 44 compounds selected from screen. **D**, Interactomic analysis using Ingenuity Pathway Analysis (IPA) software. Interactomic analysis was performed using Ingenuity Pathway Analysis (IPA) software to visualize protein networks, where proteins are depicted as hubs and their relationships as edges. This figure illustrates a network identified by IPA, highlighting direct interactions involving the 44 selected compound’s targets. Canonical pathway (CP) enriched by the targets within the network are reported **E**, Pie chart of the mechanisms of all 44 compounds selected from screen obtained interrogating DrugBank database. **F**, Dose-response curves of PDX-OS#29-C treated with a titration of the 7 primary screening-selected compounds. The Y-axis indicates the relative cell viability normalized to the untreated control. Data are mean ± SD for an experiment performed in quadruplicate. IC50 values for the 7 primary screening-selected compounds are expressed in nanomolar. R2 parameter was estimated for each single drug curve. To estimate mean and standard deviation of IC50 and R2, the concentrations of each compound were initially transformed to log (X) and normalized, and nonlinear regression was performed using the GraphPad Prism 7.0 software (GraphPad Inc.)

Considering the genetic and biological heterogeneity of OS, we decided to use one of our most aggressive cell lines, the PDX-OS#29-C, to interrogate a large MedChem library of 2880-FDA approved compounds using high-throughput screening in 384 format (Fig. 4). Twenty-four hours after seeding, cells were treated with each single dose (1µM or 0.1µM) for 4 days. Quality control and Z-f scores are shown in Suppl. Figure 2; Z analysis was rated good, ranging from 0.75 to 0.92.

Drug screenings of all drugs at the dose of 1µM identified 145 compounds that induced inhibition of OS cell growth ≥ 55% (cut-off) (Fig. 4B). Screening at the dose of 0.1µM identified 56 compounds with similar level of efficacy (Fig. 4A). Venn diagram (Fig. 4C) visualizes the 44 compounds common to both screenings. We interrogated the Ingenuity Pathway Analysis (IPA), looking for direct interactions established from the targets of the 44 drugs, extrapolated from Drug Bank database. We found that several of them closely clustered together in a network associated with “Remodeling of epithelial adherens junctions” and “Mitotic G2-G2/M phases” Canonical pathway (CP) (Fig. 4D). Moreover, the 44 drugs were clustered in 7 categories relative to their main mechanisms of action, according to Drug Bank database (Fig. 4E).

We, thus, selected 7 drugs representative of each category (homoharringtonine, clofarabine, tirbanibulin, hydroxycitric acid, ixabepilone, azlocillin, and olverembatinib) to be validated in a dose-response test using PDX-OS#29-C cells. IC_50_ values for the seven compounds selected for the screening are expressed in nM and are shown in Fig. 4F.

Dose-response analysis of the 7 drugs was expanded to the entire panel of PDX-derived cell lines using a different assay (MTT assay).

**Fig. 5:**
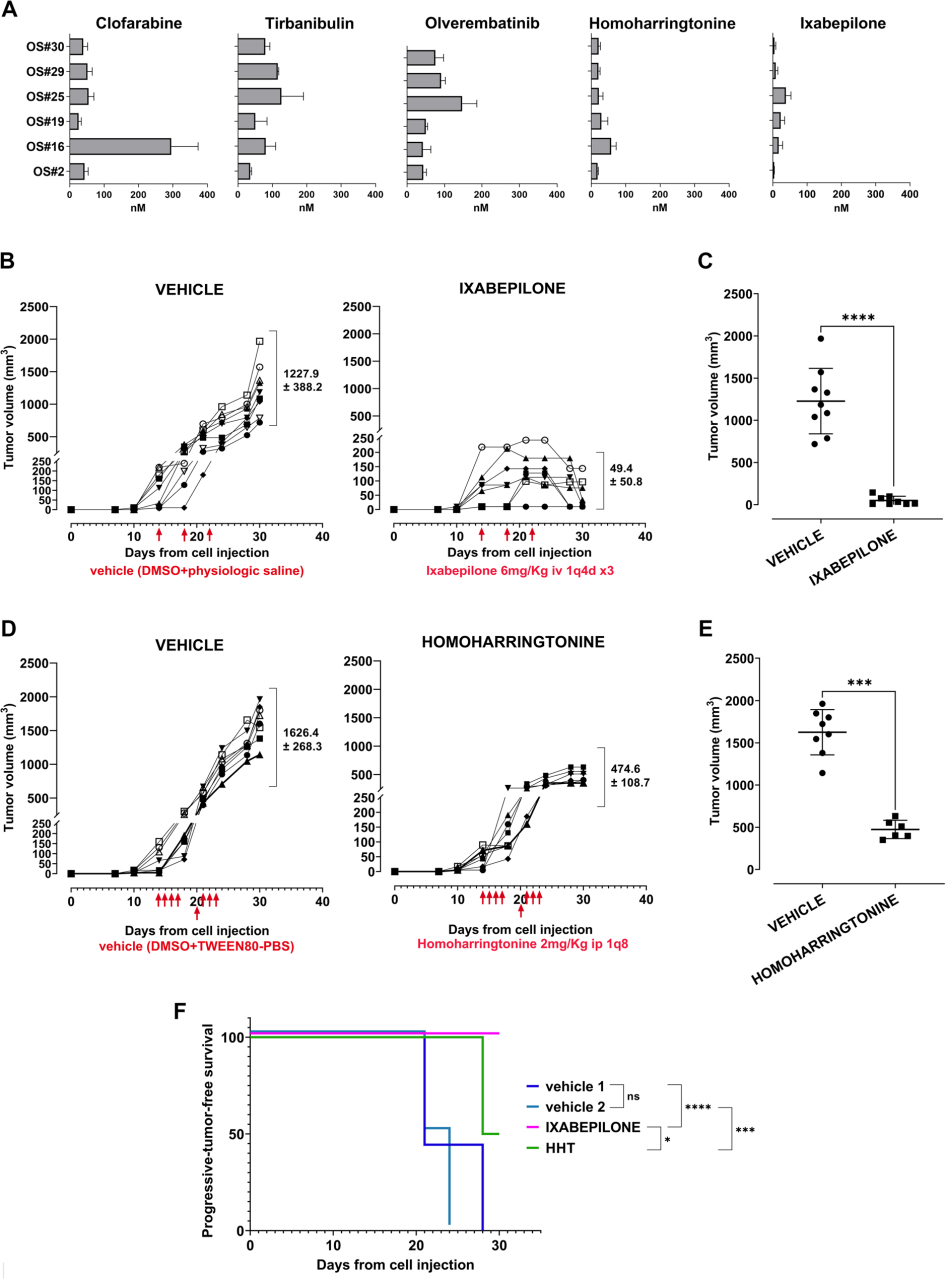
Ixabepilone showed the most effective anti-tumor activity. **A**, IC_50_ values of five out of the seven selected FDA approved drugs are shown. The IC_50_ values are expressed in nanomolar and were estimated based on the results of the MTT assay using GraphPad software. Each value represents the mean ± SD of three independent experiments. **B-C** Tumor growth in NSG mice bearing xenografts derived from the s.c. injection of the OS#30-C PDX-derived cell line after treatment with vehicle (left) or ixabepilone (right) (6 mg/kg 1q4d x3 e.v.); **B**, Growth curves for each mouse are shown. Treatment started when tumors become measurable. Arrows indicate the days of drug administration on days 14, 18, and 22 after cell injection. **C**, Tumor volumes of ixabepilone-treated mice *versus* vehicle at the end of treatment are shown. (**** Means ± SD, *p* < 0.0001, Mann Whitney U test). **D-E**, Tumor growth in NSG mice bearing xenografts derived from the s.c. injection of the OS#30-C PDX-derived cell line after treatment with vehicle (left) or homoharringtonine (right) (2 mg/kg 1q i.p. two weeks, four days/week, for a total of 8 treatments); **D**, Growth curves of each single mouse are shown. Treatment started when tumors become measurable. Arrows indicate the days of drug administration, starting from day 14 after cell injection. **E**, Tumor volumes of homoharringtonine-treated mice *versus* vehicle at the end of treatment are shown. (*** Means ± SD, *p* < 0.001, Mann Whitney U test). **F**, Kaplan–Meier progressive-tumor-free survival curves of ixabepilone and homoharringtonine treated mice compared to control groups (progressive tumor were considered over a tumor volume of 500mm^3^ at which tumor regressions were not observed), (Mantel–Cox log-rank test, ixabepilone compared to vehicle 1 (DMSO-saline i.v.) **** *p* < 0.0001; homoarringtonine compared to vehicle 2 (DMSO-PBS-Tween 80 i.p.) *** *p* < 0.001; ixabepilone compared to homoharringtonine *p < 0.05)

Only five out of the seven drugs maintained a promising profile of efficacy (ixabepilone, clofarabine, tirbanibulin, olverembatinib and homoharringtonine), while hydroxycitric acid and azlocillin failed to confirm their efficacy, with no significant inhibition at the highest concentration tested (1μM) (Fig. 5A). All the five drugs showed IC_50_ efficacy in the nM concentration range. Of particular interest resulted homoharringtonine, an inhibitor of protein translation that is FDA-approved for the treatment of chronic myeloid leukemia [42, 43], and ixabepilone, a semisynthetic analog of epothilone B with microtubule inhibitory activity that is approved for the treatment of metastatic or locally advanced breast cancer [44, 45]. Notably, both homoharringtonine and ixabepilone reduced the growth of the PDX-derived cell lines with IC_50_ values ranging from near 4 to 50nM (Fig. 5A). Drug efficacy was confirmed in other six human OS cell lines (four derived directly from patient’s tumor and two commercially available), including cell variants resistant to methotrexate and cisplatin that were previously obtained and characterized in our laboratory [22, 36] (Suppl. Table 2a), and in anchorage-independent conditions at similar nM dose range (Suppl. Figure 6). Next, we treated mice engrafted with our most aggressive PDX-derived cell line. When mice developed measurable tumors, they were divided into two cohorts and treated with either vehicles or drugs as single agent. Ixabepilone was administered i.v. at a dose of 6 mg/kg 1q4d x3, a dosage and schedule comparable to its clinical use in humans. The efficacy of ixabepilone was shown in all mice (Fig. 5B-C). Tumor volumes were significantly reduced, and tumor regression was also observed in 5 out of 8 mice. In line with other evidence [46, 47], the drug was found to be effective against a tumor that overexpresses PGP (Suppl. Figure 7), indicating that it can strongly impair the growth of chemoresistant OS. Histological analysis of representative untreated tumors (Suppl. Figure 7A) showed histological chondroblastic cell components together with undifferentiated small round, highly proliferating cells. After treatment with ixabepilone, only three mice carried measurable tumors. Histology showed that only the chondroblastic component is still present, with few if any sign of cell proliferation (Suppl. Figure 7B). Ixabepilone treatment was well tolerated in mice, with a mean nadir body weight loss of approximately 11% in the ixabepilone-treated group after the third administration, but no deaths or neurotoxicity were observed (Suppl. Figure 8A). Treatment of the mice with homoharringtonine, compared to vehicle, also significantly reduced tumor growth (Fig. 5D-E) but without any evidence of tumor regression. Both ixabepilone and homoharringtonine significantly prolonged the survival of mice (Fig. 5F), but the efficacy of ixabepilone was remarkably higher. The treatment with homoharringtonine was also well tolerated in mice (Suppl. Figure 8B).

## Discussion

Over the past decade, significant progress has been made in understanding the genetic landscape of OS, offering insights into potential therapeutic targets. However, identifying agents with durable efficacy for patients with relapsed or metastatic OS remains a chimera. This challenge is partly due to the tumor’s complexity and limitations in previous preclinical studies, which failed to adequately model the genetic heterogeneity and plasticity of OS or effectively characterize resistant subpopulations within individual tumors. The current availability of well-characterized patient-derived models provides better options to identify promising treatments that match the molecular make-up of the tumor [8, 48]. However, considering the huge OS genetic heterogeneity, as many PDXs or cell lines as possible are needed to best model OS. We contribute to the collection of PDXs from OS, by presenting models that cover the etiological, histological, and genetic diversity of OS and maintain the features of the original tumors. Our collection includes treatment naïve OS models as well as drug refractory and metastatic ones, including two longitudinal models (tumor models generated from the same patient using tumors obtained at various timepoints along the therapy continuum). Particularly, from one of these two longitudinal models we obtained matched PDX-derived cell lines, which are representative of a naïve tumor with innate features of chemoresistance and a post-chemo tumor unresponsive to current standard therapies for OS. We believe that these two cell lines, together with four others obtained from PDXs representative of OS that led patients to death, are valuable tools to obtain insight into resistance mechanisms and to prioritize new drugs. The six PDX-derived cell lines were injected into NSG mice to study local tumor growth and metastasis. Matched patient samples, PDX, PDX-derived cell lines and xenografts grown after injection fo PDX-derived cell lines (CDXs) were molecularly characterized using targeted genome sequencing and digital PCR. All the major genetic alterations were maintained in paired models, including the xenografts developed from cell lines, representing a collection of well annotated experimental models that will help our understanding of therapy sensitivity *versus* resistance over time and across OS subtypes.

The PDXs and PDX-derived cell lines showed multiple and peculiar genetic alterations. Frequency of the major molecular alterations are consistent with those reported by others [8, 41] *TP53* missense mutations and *MYC* gene amplification were the most common. In line with Schott CR et al. [41], we were unable to correlate a specific set of genetic alterations with the heterogeneous behavior of the OS cell lines in terms of local tumor growth and metastasis. However, the most aggressive experimental models were the two paired cell lines that derived from a chondroblastic OS (before and after chemotherapy). The two cell lines showed gain of two major oncogenes (*MYC*, *CCNE1*), loss of the oncosuppressor *RB1*, overexpression of PGP and developed fast growing local tumors and lung metastasis in 100% of the animals, thus modeling a very aggressive subtype of OS. Such highly malignant behavior was in line with the poor clinical history of the patient who died for disseminated disease within 8 months from diagnosis. We thus decided to use the highly aggressive PDX-OS#29 for high-throughput drug screening of a library including 2880 FDA-approved compounds to prioritize compounds that could have efficacy against the subgroup of very aggressive, chemo-resistant OSs. The development of new drugs for rare tumors, such as OS, is a major challenge. Indeed, considering that the process of developing a novel drug to treat any kind of disease is typically laborious, costly, and failure-prone, development of new agents for rare tumors is particularly unappealing for large manufacturers. In addition, for the few agents that are developed, market prices are usually extremely high, resulting in reduced access for patients. In this context, drug reuse is an appealing and promising approach to develop medicines for uncommon diseases, including rare tumors [49]. By utilizing already available compounds with deep knowledge of pharmacological characteristics, action mechanisms, and safety profiles, drug repurpose may indeed speed up the inclusion of novel drugs for the treatment OS patients. Our high-throughput drug screening identified 44 compounds grouped into 7 categories in relation to their main mechanisms of action. The efficacy of seven drugs, representative of the 7 categories, was also evaluated against the other PDX-derived cell lines to obtain a more general information. Five out of seven drugs were found to be active at clinically acceptable doses. Homoharringtonine, a protein synthesis inhibitor that was also reported to deregulate MYC expression [43] and ixabepilone, a semisynthetic analog of epothilone B with microtubule inhibitory activity [19] were found to be the most potent drugs. Both the two drugs were active at nM concentrations in vitro against all the 12 human OS cell lines here considered (PDX-, human tumor-derived, commercial cell lines), and they maintained their efficacy against chemo-resistant cell variants established in our laboratory [22, 36]. However, experiments in mice clearly showed a superior activity of ixabepilone compared to homoharringtonine. Indeed, ixabepilone led to tumor regression in the majority of the mice bearing an aggressive, chemoresistant OS, while homoharringtonine induced a prevalent cytostatic effect on tumor growth. In the three mice that carried a small OS mass after treatment with ixabepilone, the tumor resulted to be encapsulated into the muscle tissues and composed of chondroblast-like cells, without recognition of cell proliferation. In addition, the progressive-tumor free survival of mice treated with ixabepilone was higher compared to mice treated with homoharringtonine.

Of importance is that both in vitro and in vivo preclinical studies, ixabepilone is reported to retain cytotoxic activity in taxane- and anthracycline-resistant tumor cells [50]. Preclinical studies in taxane- and anthracycline-resistant breast cancer cell lines have demonstrated that ixabepilone displays a higher affinity for β-tubulin compared to taxanesand it is not a substrates of PGP [46]. In addition, ixabepilone exhibits a broader spectrum of antitumor activity in preclinical models of childhood tumors, including OS, than paclitaxel, which proved to be clinically inactive in childhood cancers [51]. Not surprisingly, these early results showed higher sensitivity of rabhoid, brain and Wilm’s tumors to ixapebilone compared with OS, which remains a tumor type that is difficult to be modelled and treated. However, some complete responses were observed when 6 OS PDXs were treated with 10–15 mg/kg of ixabepilone, substantially confirming our positive results. At clinical level, Ixabepilone has a manageable toxicity profile [44, 52] and is an FDA-approved therapeutic option for patients with metastatic breast cancer that progressed despite prior anthracycline and taxane therapy. It is also approved in combination with capecitabine for patients with triple-negative breast cancer, who have limited treatment options after failing previous therapies [19]. The activity of this drug was assessed in a phase II trial within the Children’s Oncology Group [53], based on the safety and tolerability data from a Phase I study [54].The study reported negative results. However, inconsistencies between preclinical and clinical studies may be due to several factors, including the different dosing schedule used in preclinical studies compared to the phase II trial, the fact that clinical trial did not consider prolonged stable disease as primary end-point, the possible different effect of the drug in adults and children, and the fact that the study was conducted in a heavily pretreated patient population. In addition, ixabepilone was administered without any consideration of the molecular profile of the tumors. In this paper, we support the idea that the drug is quite effective in aggressive, chemoresistant OS, showing multiple genetic alterations and overexpression of P-glycoprotein.

Considering that PGP is known to be overexpressed in approximately 40% of OS at diagnosis and its presence is associated with multidrug resistance and worse prognosis [34, 40] our study supports the incorporation of ixabepilone into treatment plans for OS who are resistant to currently available agents and challenges to re-consider this drug for future treatment strategies in selected high-risk OS groups.

## Conclusions

Patients with highly aggressive, chemoresistant osteosarcoma (OS) continue to represent a population with unmet need. Historically there has been a limited number of relevant models that well represent the heterogeneity of OS to be used for discovering efficient novel drugs. We have enlarged the already available collections of patient–derived xenograft (PDX) with 30 additional models that depict the epidemiologic, genetic, and biological features of OS and we have established six, new PDX-derived cell lines as a resource to generate solid preclinical findings to guide treatments for patients with these aggressive malignancies. Drug screening of 2800 FDA-approved compounds led to identification of five repurposed drugs, including ixabepilone that was selected as the most effective agent against highly aggressive OS with features of innate and/or acquired chemoresistance. Overall, our work will enrich the scientific community with other in vitro and in vivo aggressive OS models to be used for testing novel therapies and support the use of an already existing medicine for the treatment of an OS patient population that continues to represent an unmet need.

## Electronic supplementary material

Below is the link to the electronic supplementary material.

Supplementary Material 1

Supplementary Material 2

Supplementary Material 3

Supplementary Material 4

Supplementary Material 5

Supplementary Material 6

Supplementary Material 7

Supplementary Material 8

Supplementary Material 9

Supplementary Material 10

Supplementary Material 11

## Data Availability

All sequencing data that support the findings of this study are publicly available in Sequence Read Archive (SRA) at BioProject and are accessible through the accession number PRJNA1208251. The PDXs, PDX-derived cell lines, and CDX data generated in this study are available upon request from the corresponding author. PDXs and PDX-derived cell lines, not available through public repositories, are available on request to the corresponding authors and through material transfer agreement. All other data are present in the main text or in the Supplementary Data.

## Abbreviations

AYA: Adolescents/young adults
BWA: Burrows-Wheeler Alignment
CDX: Cell-derived-xenograft
CNV: Copy number variant
CO_2_: Carbon dioxide
CP: Canonical pathway
ddPCR: Droplet digital PCR
DMSO: Dimethyl Sulfoxide
EDTA: Ethylenediaminetetraacetic acid
FBS: Fetal bovine serum
FDA: Food and drug administration
GC: Guanine-cytosine
HHT: Homoharringtonine
hPBMC: Peripheral blood mononuclear cells
IMDM: Iscove’s modified Dulbecco’s medium
IPA: Ingenuity Pathway Analysis
IXA: Ixabepilone
MBC: Molecular barcode
MDR-1: Multidrug resistant-1
NSG: NOD scid gamma
OS: Osteosarcoma
PCR: Polymerase chain reaction
PDX: Patient-derived xenograft
PDX-C: Patient-derived-xenografts cell line
PGP: P-glycoprotein
SNV: Single nucleotide variant
STR: Short tandem repeat
VEP: Variant Effect Predictor
