# Supplementary figures and images for: Genomic profiling of a collection of patient-derived xenografts and cell lines identified ixabepilone as an active drug against chemo-resistant osteosarcoma
Source: J Exp Clin Cancer Res. 2025 Jul 8;44:195. doi: 10.1186/s13046-025-03440-5 (PMC12235892; doi:10.1186/s13046-025-03440-5)

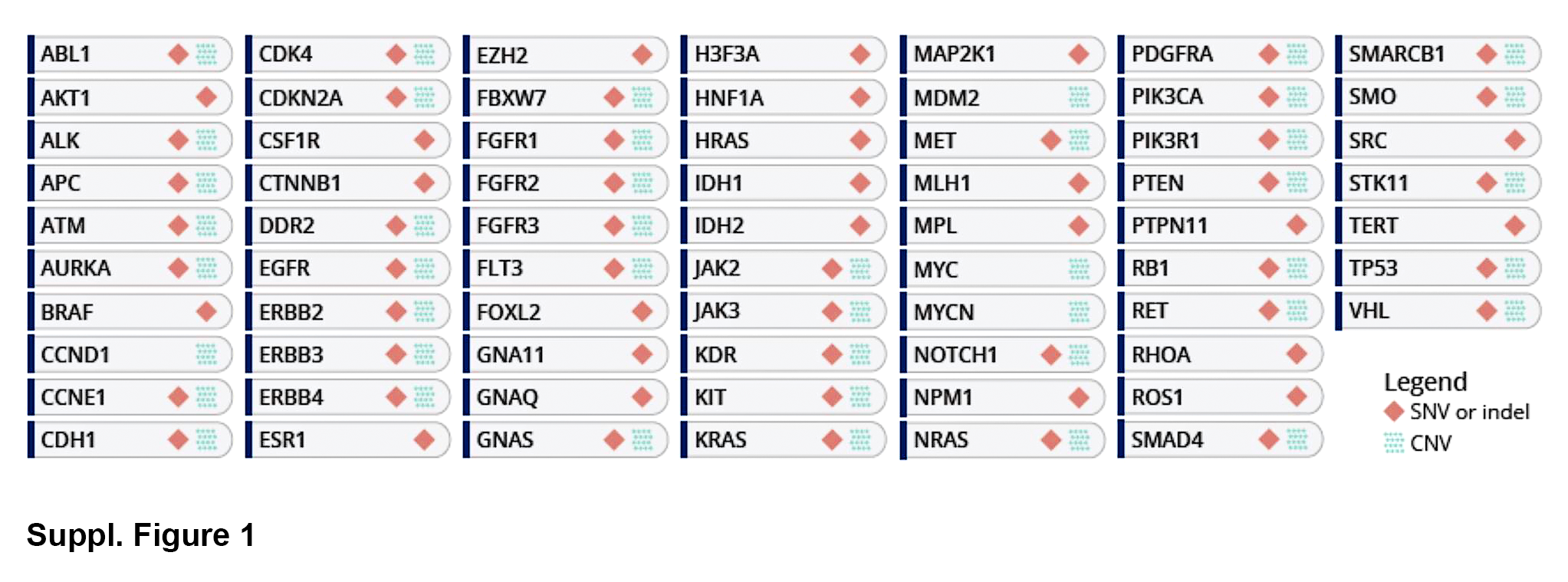

Supplement: Supplementary file 1 — Supplementary Material 1 [file 13046_2025_3440_MOESM1_ESM.tif]

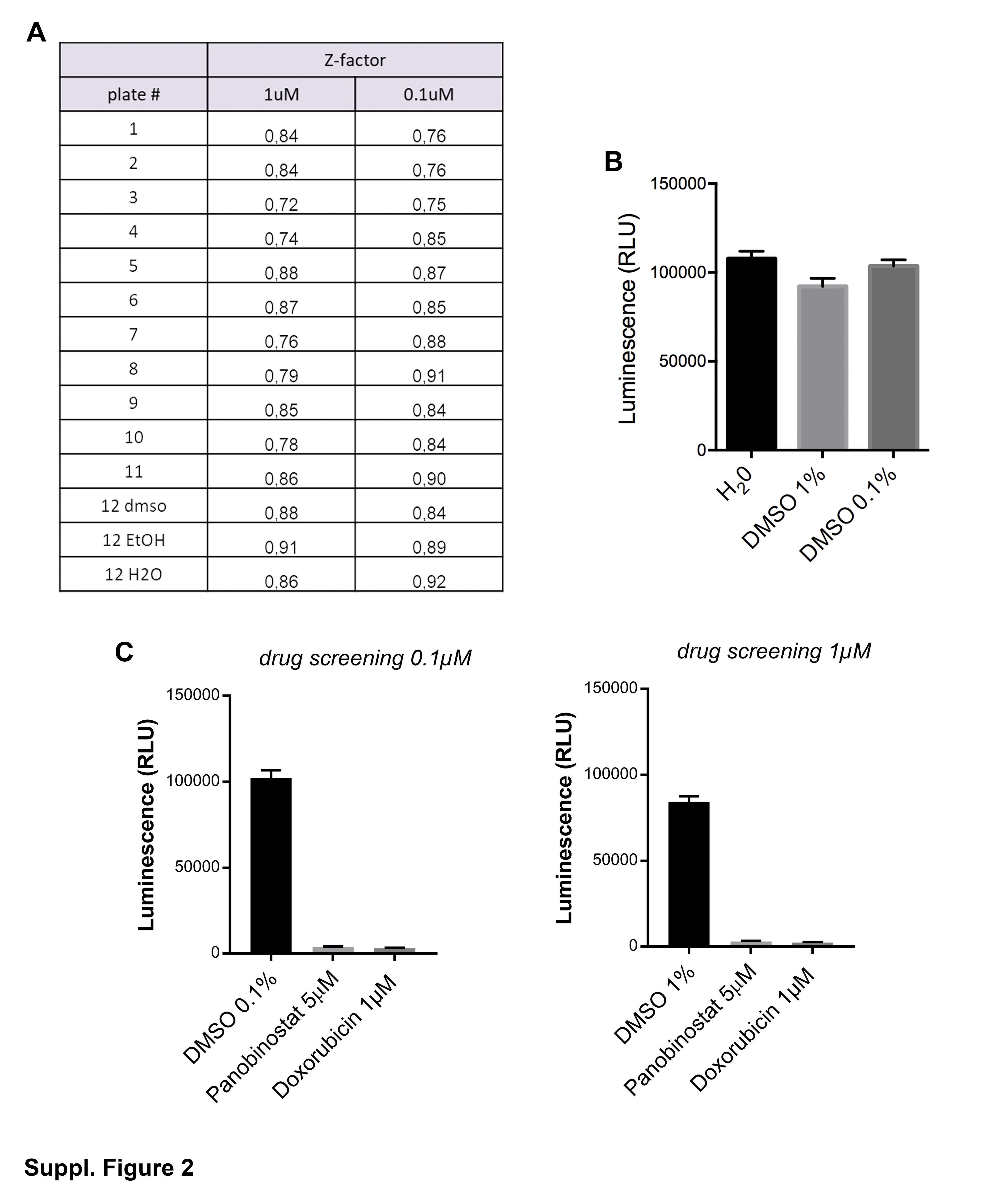

Supplement: Supplementary file 2 — Supplementary Material 2 [file 13046_2025_3440_MOESM2_ESM.tif]

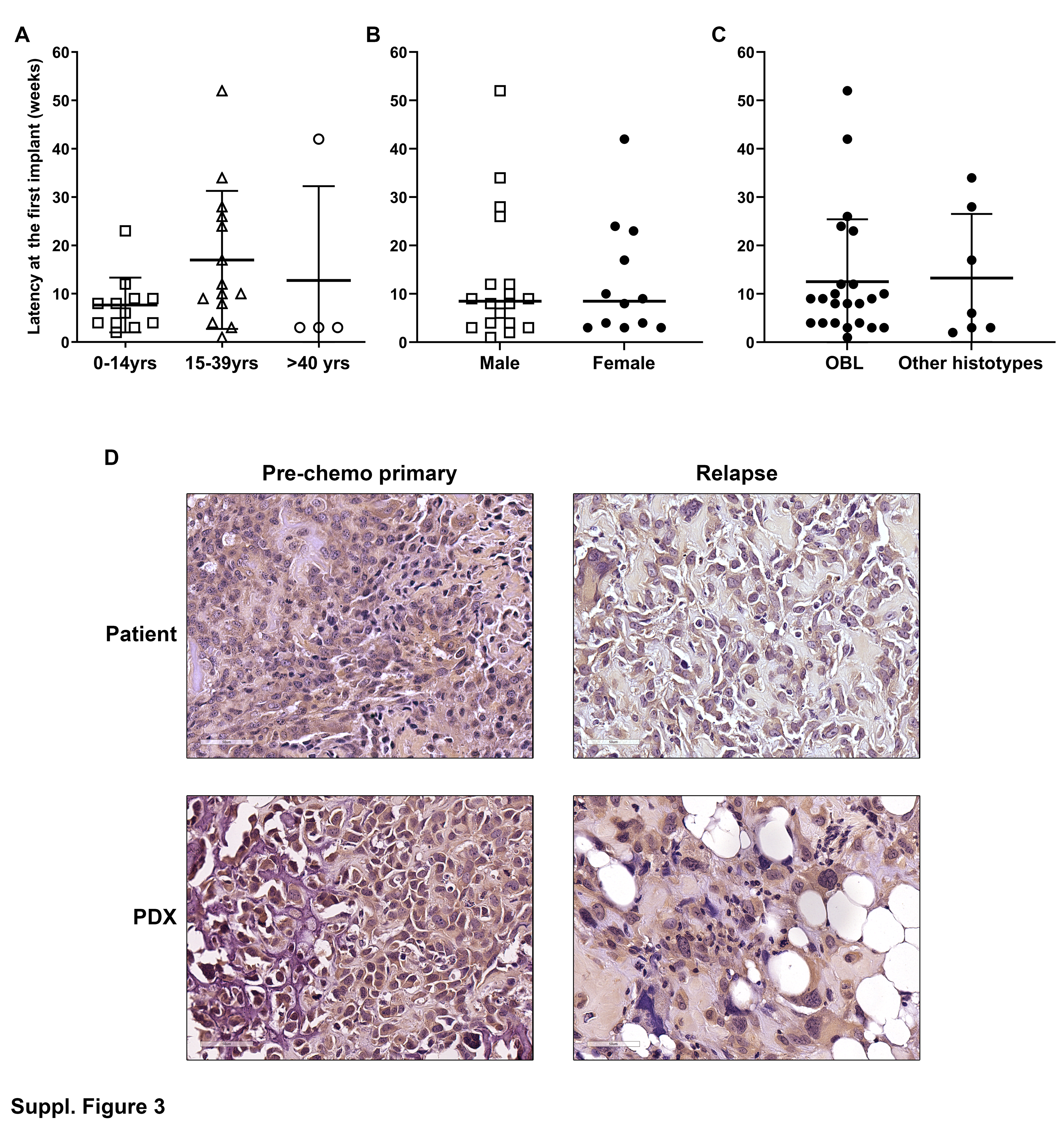

Supplement: Supplementary file 3 — Supplementary Material 3 [file 13046_2025_3440_MOESM3_ESM.tif]

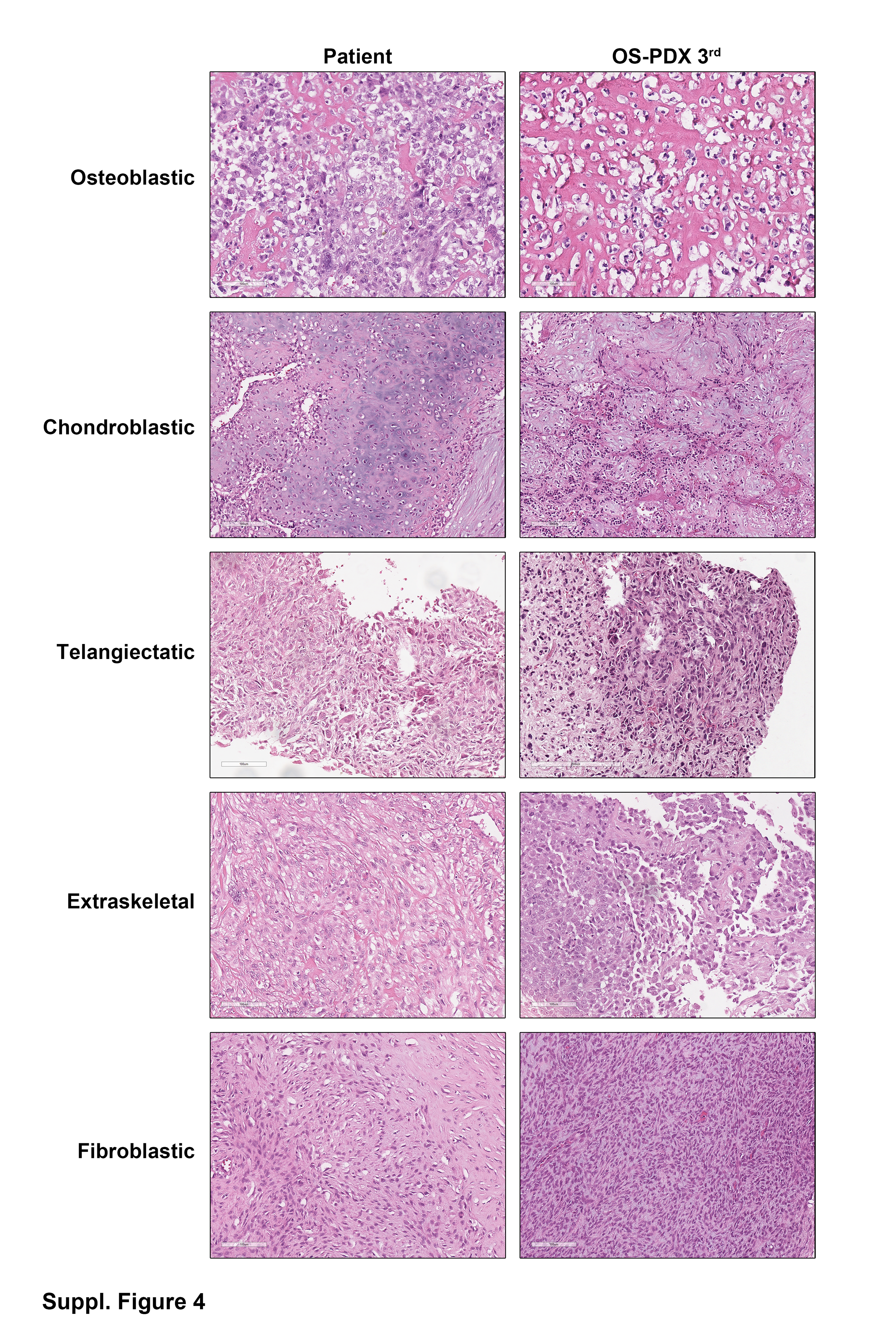

Supplement: Supplementary file 4 — Supplementary Material 4 [file 13046_2025_3440_MOESM4_ESM.tif]

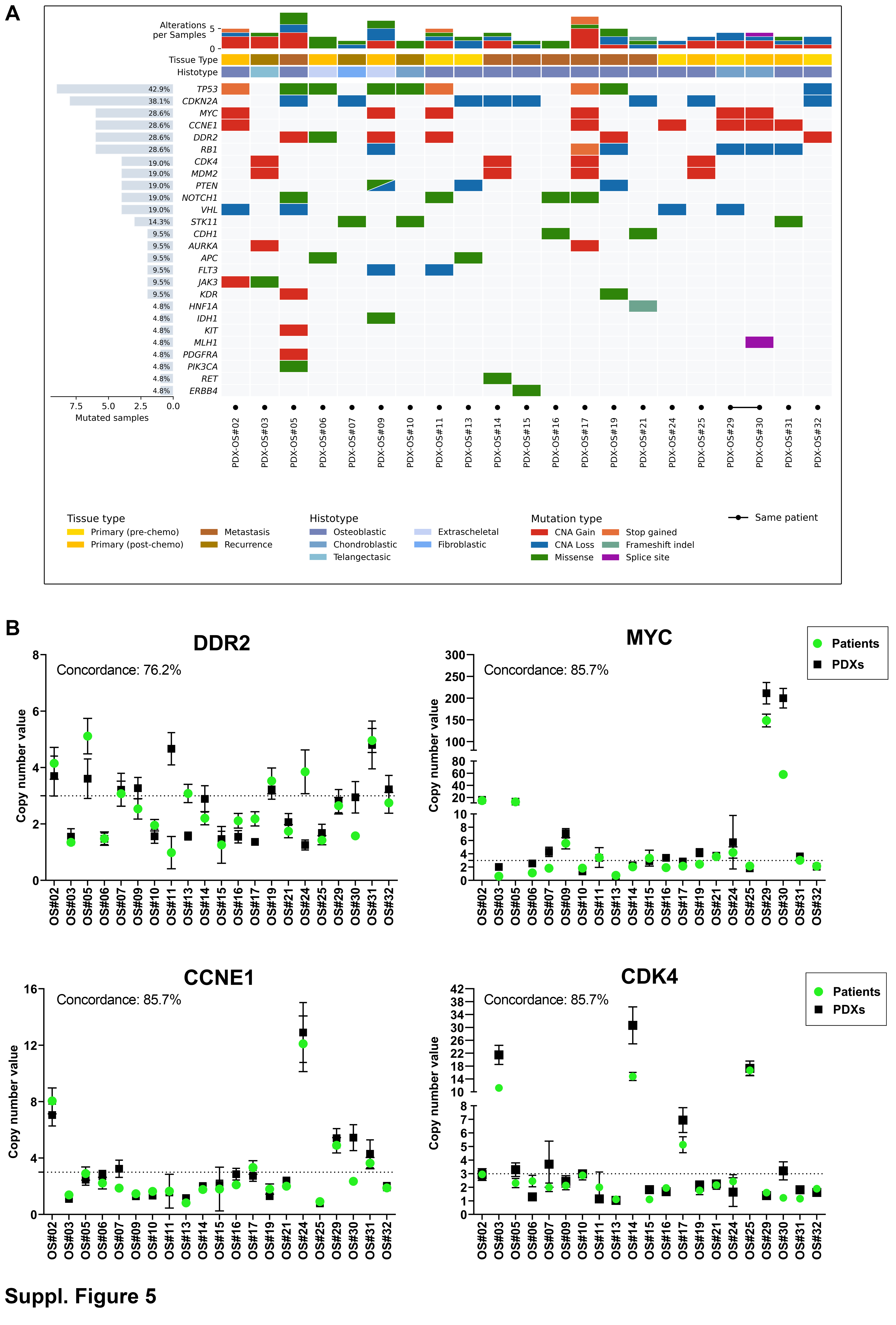

Supplement: Supplementary file 5 — Supplementary Material 5 [file 13046_2025_3440_MOESM5_ESM.tif]

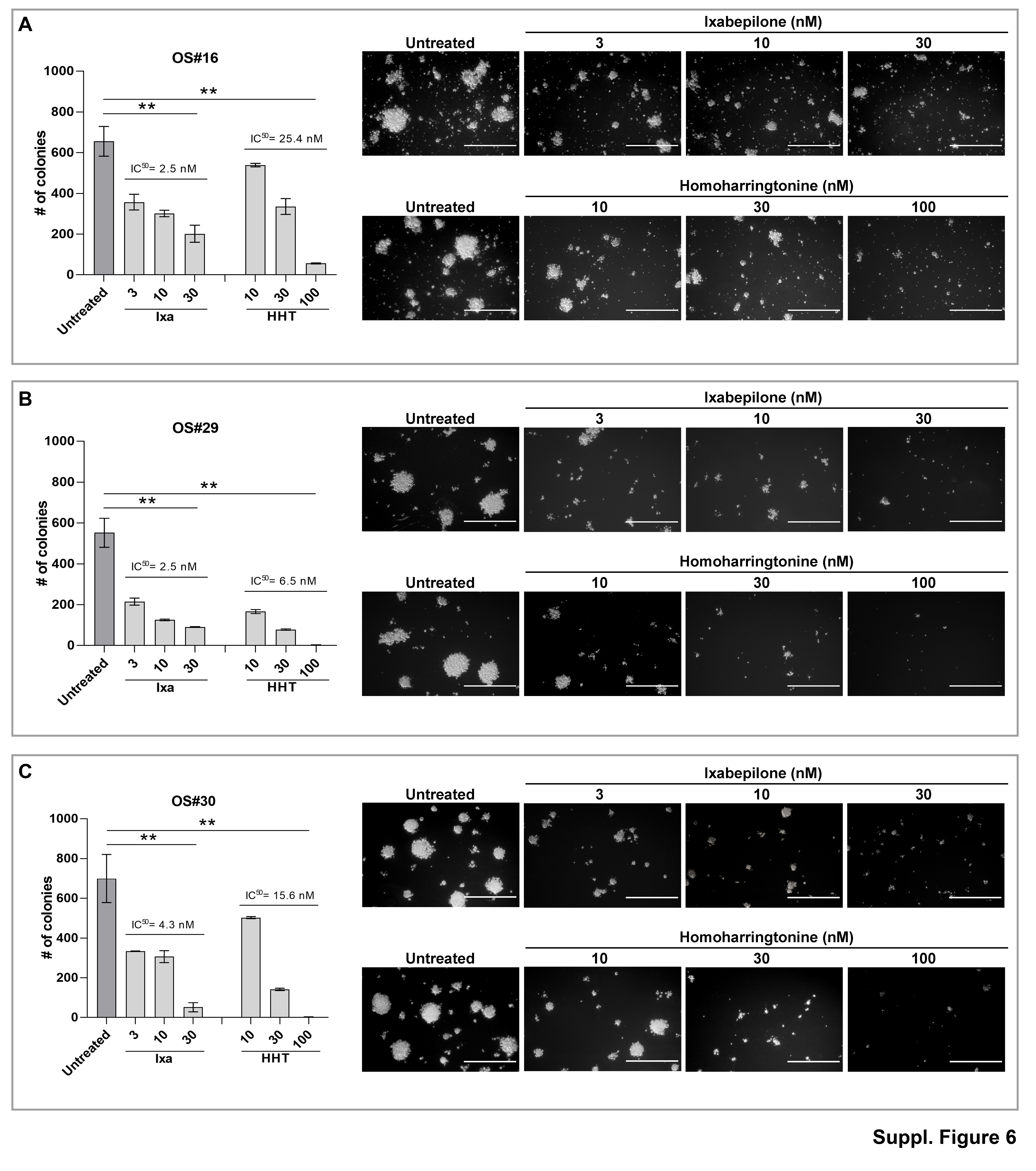

Supplement: Supplementary file 6 — Supplementary Material 6 [file 13046_2025_3440_MOESM6_ESM.tif]

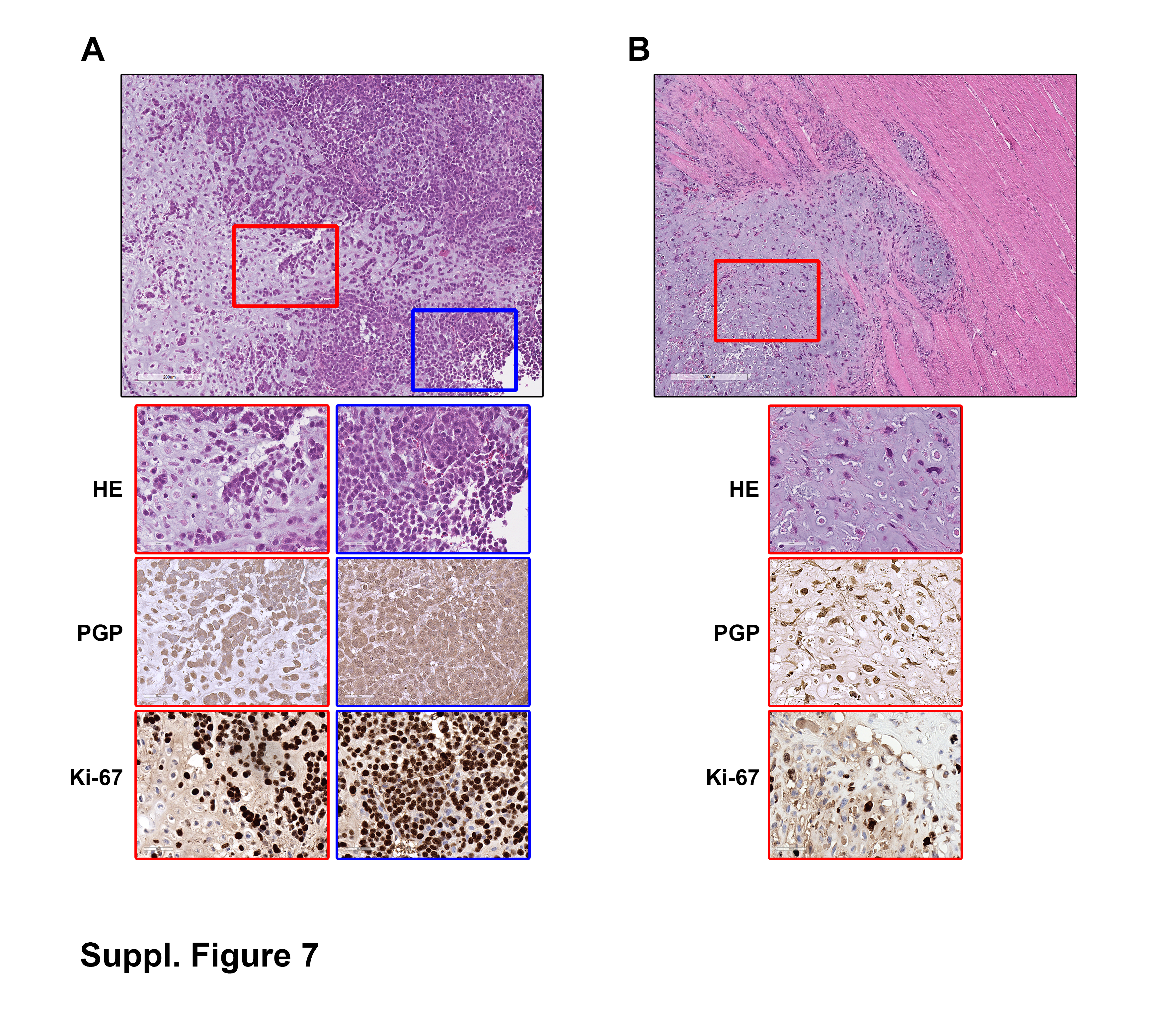

Supplement: Supplementary file 7 — Supplementary Material 7 [file 13046_2025_3440_MOESM7_ESM.tif]

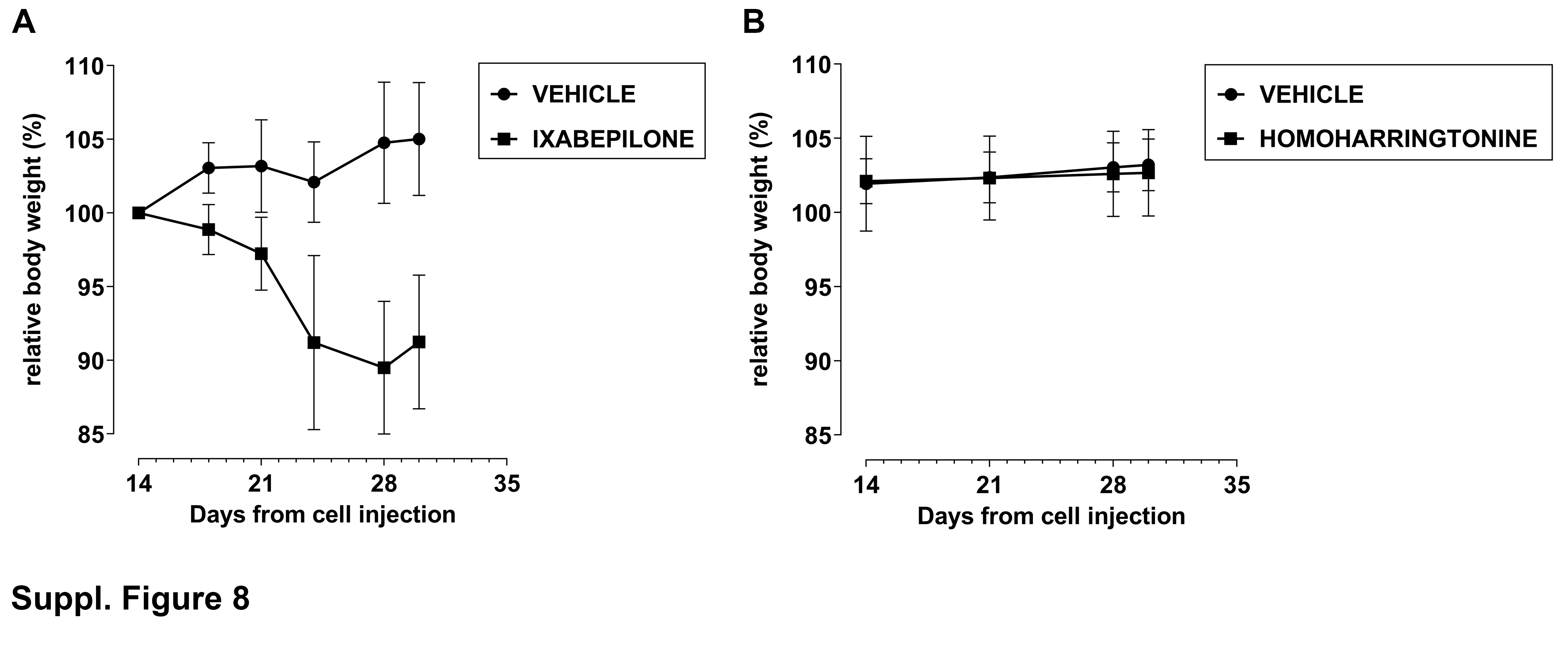

Supplement: Supplementary file 8 — Supplementary Material 8 [file 13046_2025_3440_MOESM8_ESM.tif]
