# Supplementary material for: Genomic profiling of a collection of patient-derived xenografts and cell lines identified ixabepilone as an active drug against chemo-resistant osteosarcoma
Source: J Exp Clin Cancer Res. 2025 Jul 8;44:195. doi: 10.1186/s13046-025-03440-5 (PMC12235892; doi:10.1186/s13046-025-03440-5)
Supplement: Supplementary file 9 — Supplementary Material 9 [file 13046_2025_3440_MOESM9_ESM.docx]

**Supplementary Figure legends**

**Supplementary Figure 1.** Archer® VariantPlex® Solid Tumor panel, 67 genes associated with solid tumors.

**Supplementary Figure 2. High-throughput drug screening quality parameters. A**, Z-factor values calculated for each plate during the HTS performed at 0.1μM and 1μM. **B**, Representative negative controls showing the no toxic effects of the solvents alone in which some compounds have been dissolved. c. Cells shown high sensitivity to cytotoxic drugs such as doxorubicin 1 μM and panobinostat 5 μM, used as positive controls. Negative controls and positive controls are evaluated in each single plate treated during the drug screening at 0.1μM and 1μM.

**Supplementary Figure 3. PDX tumor latency and sample characteristics. A,** PDX tumor latency at first implant and patient age (no significant differences by one-way ANOVA nonparametric tests and Kruskal-Wallis and Dunn post hoc tests). **B,** PDX tumor latency at first implant and patient gender (no significant differences by Mann-Whitney U test). **C,** PDX latency at first implant and tumor histology (no significant differences by Mann-Whitney U test). **D,** Representative images of immunohistochemical staining for P-glycoprotein expression in patient tumor samples (upper panels) and paired PDXs (lower panels) of a pre-chemo (*left*) and a metastatic (*right*) specimen. Scale bar 50 μm.

**Supplementary Figure 4. OS-PDXs recapitulate key histologic features of the parental tumor of origin.** Histological features of patients’ tumors and corresponding PDX at the third generation. Sections were stained with hematoxylin and eosin (H&E) PDXs closely resembled patient’s tumor, including the production of neoplastic bone and the presence of anaplastic cells. Bar: 100 μm.

**Supplementary Figure 5. Genomic characterization of PDX samples. A,** Heatmap visualization of alterations in cancer-related genes in a cohort of 21 PDX-OS samples from 20 OS patients. Copy number gain/loss was defined as log2 ratio segments log2 >1.7 and log2<-1.7, respectively. The upper plot summarizes alterations per sample and clinicopathologic characteristics of the samples. The left bar graph shows the percentage of altered genes PDX-OS samples. At the bottom of the plot, samples from the same patient are labeled as linked circle indicators. Each point represents a single sample. **B**, Determination of copy number variation (CNV) status in OS patients and OS PDX models by ddPCR for DDR2, MYC, CCNE1 and CDK4. Each marker represents a CNV measurement from a single ddPCR well of ∼ 20,000 drops. Error bars indicate Poisson 95% confidence intervals for each copy number determination. Amplification was defined as the detection of CNV >3 gene copies. The percentage of genetic concordance between paired patient and PDX models was reported.

**Supplementary Figure 6.** Colony formation assays in soft agar of PDX-OS cells exposed to various concentrations of ixabepilone (range 3-30 nM) and homoharringtonine (range 10-100 nM). Data are represented as mean ± SD (n=3). ** p<0.01, Kruskal-Wallis test. The IC50 values are expressed in nanomolar and were estimated based on the results of the spheroid counts using GraphPad software. Representative images are included. Bar: 500 µm.

**Supplementary Figure 7.** Representative H&E images of CDXs from OS#30-C cells treated **(B)** or not **(A)** with ixabepilone (scale bar 200µm). The enlarged box shows morphology, PGP, and Ki-67 expression in untreated versus ixabepilone-treated tumors.

**Supplementary Figure 8.** Weight variation in NSG mice following *in vivo* treatment with ixabepilone **(A)** and homoharringtonine **(B)**
