# Supplementary material for: Genomic profiling of a collection of patient-derived xenografts and cell lines identified ixabepilone as an active drug against chemo-resistant osteosarcoma
Source: J Exp Clin Cancer Res. 2025 Jul 8;44:195. doi: 10.1186/s13046-025-03440-5 (PMC12235892; doi:10.1186/s13046-025-03440-5)
Supplement: Supplementary file 11 — Supplementary Material 11 [file 13046_2025_3440_MOESM11_ESM.docx]

**Supplementary Table 2a**

Osteosarcoma patient-derived cell lines, patient characteristics, genomic alterations and biological features.

| Cell  line ID | Patient | | | | Cell line | | | | | | |
| --- | --- | --- | --- | --- | --- | --- | --- | --- | --- | --- | --- |
|  | **Age** | **Sex** | **Met** | **status** | **Origin** | **Tx**  **staus** | **Site**  **origin** | **Genomic**  **Features*** | **PGP % of positive cells (MFI)** | **Spheroids**  **(soft agar)** | **DXR IC50 (ng/ml)** |
| U-2OS | 15 | female | unknown | unknown | primary | treated | bone | *CDKN2A del* | 15.6 (1.5) | 1748 ± 489 | 39.2±26.1 |
| SaOS-2 | 11 | female | unknown | unknown | metastasis | treated | lung | *p53 del* | 0.8 (0.4) | 773 ± 184 | 125.6±11.0 |
| IOR/OS9 | 15 | male | yes | dead | metastasis | treated | bone | *CDKN2A del* | 0 (0) |  | 25.4±4.1 |
| IOR/OS10 | 10 | female | no | alive | primary | treated | bone | *CDKN2A del, p53 mut* | 2.4 (3.7) | 75 ± 30 | 6.0± 2.0 |
| SARG | 25 | male | yes | dead | primary | naive | bone | *CDKN2A del, p53 mut* | 5 (2.6) | 777 ± 93 | 65.6±20.6 |
| MOS | 13 | female | yes | dead | primary | naive | bone | *p53 del* | 0.8 (0.1) | 243 ± 97 | 192.3±18.8 |

*(*) data from Ottaviano L, et al Genes Chromosomes Cancer. 2010 Jan;49(1):40-51. doi: 10.1002/gcc.20717)*

**Supplementary Table 2b**

IC_50_ values of patient-derived OS cell lines (IOR/OS9, IOR/OS10, SARG and MOS), two commercial cell lines (SaOS-2 and U-2OS) and U-2OS cell resistant variants to methotrexate and cisplatin (U-2OS/MTX300 and U-2OS/CDDP4ug) of ixabepilone and homoharringtonine are shown. The IC_50_ values are reported in nanomolar concentrations and were estimated based on the results of the MTT assay using GraphPad software. Each value represents the mean ± SD of three independent experiments.

| **Cell**  **line ID** | **Ixabepilone**  **(mean IC_50_ ± SD, nM)** | **Homoharringtonine (mean IC_50_ ± SD, nM)** |
| --- | --- | --- |
| **SaOS-2** | 9.8± 0.6 | 17.6± 1.3 |
| **IOR/OS9** | 6.4± 0.4 | 28.6± 3.6 |
| **IOR/OS10** | 2.7± 0.1 | 20.8± 2.5 |
| **SARG** | 3.2± 0.1 | 16.4± 1.2 |
| **MOS** | 19.4± 5.0 | 53.4± 1.2 |
| **U-2OS** | 12.0± 2.1 | 17.4± 1.3 |
| **U-2OS/MTX300** | 16.1± 8.2 | 17.0± 3.3 |
| **U-2OS/CDDP4µg** | 14.2± 5.0 | 21,3± 4.4 |
